# Aromatic residue-rich amino-terminal segments of temporin L self-assemble into collagen-mimetic peptides with cell-adhesion properties

**DOI:** 10.1016/j.jbc.2026.111356

**Published:** 2026-03-10

**Authors:** Neeraj Kumar Verma, Arvind Gupta, Malika Arora, Nabanita Mukherjee, Tayyaba Afshan, Rahul Dev Verma, Rahul Verma, Jyotshana Saroj, Garima Pant, Sariyah Akhtar, Surajit Ghosh, Kalyan Mitra, Deepa Ghosh, Jimut Kanti Ghosh

**Affiliations:** 1 Biochemistry and Structural Biology Division, CSIR-Central Drug Research Institute, Lucknow, India; 2 School of Studies in Biotechnology, Shaheed Mahendra Karma Vishwavidyalaya, Bastar, Jagdalpur, Chhattisgarh, India; 3 Academy of Scientific and Innovative Research (AcSIR), Ghaziabad, India; 4 Chemical Biology Unit, Institute of Nano Science and Technology, Mohali, Punjab, India; 5 Smart Healthcare, Interdisciplinary Research Platform, Indian Institute of Technology, Jodhpur, Rajasthan, India; 6 CSIR-Indian Institute of Toxicology Research, Vishvigyan Bhawan, Lucknow, Uttar Pradesh, India; 7 Electron Microscopy Unit, CSIR-Central Drug Research Institute, Lucknow, Uttar Pradesh, India

**Keywords:** cell-adhesion property, collagen-mimetic peptides, hydrogel, nanofibrils, self-assembling peptides, Temporin L, triple helical structure

## Abstract

Intriguingly, 13-mer frog-peptide, temporin L (TempL) contains 50% aromatic residues within its first eight residues. Considering the role of aromatic residues in self-assembly of peptides and the potential of such peptides in biomedical applications, we envisaged to identify new short self-assembling peptides from the amino-terminus of TempL and characterize their structural and biological properties. Thus, starting from the eighth to the first residue of TempL, we synthesized five 4 to 8 residue peptides (T-4mer to T-8mer). Different ultrastructural studies suggested nano-spherical/nano-fibrillar structures of these peptides. Remarkably, T-6mer, T-7mer and T-8mer exhibited polyproline type-II circular dichroism spectra of collagen-like triple-helical structure and sigmoidal melting curves like that we observed with rat-tail type-I collagen. Amazingly, the T-8mer peptide at 1.5% (w/v) forms hydrogel within an hour indicating its ability to form supramolecular assembly, saturated with water. We further studied collagen-mimetic nature of these TempL-derived peptides. HepG2 cells showed significant adhesions onto the coatings of T-6mer, T-7mer, T-8mer peptides and rat-tail type-I collagen which got compromised when these cells were pre-treated with antibody of collagen receptor, integrin α2β1. Interestingly, following the adhesions onto the surface of these TempL-derived peptides, cytoskeletal organization was induced in HepG2 cells like that observed in the presence of a collagen protein. Overall, the current results demonstrated the dissection of a frog-peptide, TempL with revelation of collagen-mimetic peptides from its aromatic-residue rich amino-terminus.

Self-assembling peptides demonstrate various biomedical applications (1, 2, 3, 4). Therefore, identification of new self-assembling peptides with biological activities could be of significant value. Peptides with self-assembling properties are attractive within the same family because of their diverse amino acid composition, biocompatibility and biodegradability. Advantageously the self-assembling properties of the peptides could be modulated by integration of various functional domains by combining different hydrophobic and hydrophilic amino acid residues in a single building block (5, 6). Peptides with different secondary structures such as α-helix, β-sheet and polyproline type-II (PPII) helix further organize into highly ordered supramolecular structures, including nanospheres, nanofibers, nanotubes and hydrogels (7).

Considering the usefulness of self-assembling peptides in various biomedical applications (3, 8) we looked for identifying new self-assembling peptides in naturally occurring peptides. 13-mer frog-antimicrobial peptide, TempL is considered as a lead molecule for antimicrobial-drug development for its short size and versatile antimicrobial properties (9, 10). A careful look into the amino acid sequence of TempL indicates that it possesses an abundant number (4/13, ∼25%) of aromatic residues. Surprisingly, these aromatic residues are located within its first eight residues from the N-terminus to the central region. While its C-terminus, from the 9th to 13th residue comprises of only aliphatic amino acid residues.

Aromatic stacking interactions play a crucial role in the self-assembly of peptides and these residues are often found in self-assembling peptides (11, 12). The high proportion (4/8, 50%) of aromatic residues within the first eight residues at the N-terminus of TempL lead us to explore the identification of new self-assembling scaffolds by synthesizing several peptides from this region of the peptide despite there is no report on its self-assembling property. An earlier study suggested that hydrophobic aromatic groups could drive collagen like supramolecular self-assembly in model peptides (13). A 32-mer model peptide, (glycine proline hydroxyproline)_10_ with phenylalanine and pentafluorophenylalanine at its two ends yielded favorable stabilization energy for interaction between the pairs of triple helices which self-assembled into micrometer-scale fibrils and showed the biological activity of collagen (13).

None of the synthesized TempL-derived peptides possesses any amino acid signature of collagen or the reported collagen-mimetic peptides in the literature. However, considering the abundance of the aromatic residues and their interesting positions in these designed peptides, we sought to investigate the structural and biological properties of these TempL-derived peptides in the quest of new biologically-active self-assembling peptides. As described in the following section that our current investigation revealed the identification of three collagen-mimetic peptides from aromatic-residue rich amino-terminus of TempL. One of these peptides, namely the 8-mer peptide, T-8mer readily formed hydrogel in aqueous environment and also demonstrated appreciable mechanical properties. Further, we have designed a new variant of T-8mer which appreciably retained the nano-fibrillar assembly and cell-adhesion properties of its parent collagen-mimetic peptide.

## Results

### Consideration of amino-terminus of frog antimicrobial peptide, Temporin-L for revelation of new self-assembling peptides

Besides the four aromatic residues, namely three phenylalanine residues and a tryptophan residue, the first eight residues of TempL also contain a glutamine, a serine and a valine residue (Table 1) that also occur in self-assembling peptides. Thus the N-terminus of TempL possesses significant primary structure for exploring the identification of new self-assembling peptide scaffolds. Considering the ability of the aromatic amino acid residue at the two ends of a peptide chain to promote its supramolecular self-assembly which has already been mentioned (13), we have fixed the carboxy terminus of all our synthesized peptides with a phenylalanine residue at the eighth position TempL. Since the chemical synthesis of a peptide proceeds from the carboxy-to the amino-terminus, we first synthesized a 4-mer peptide (T-4mer) which comprises the amino acid residues from the eighth to the fifth position of TempL. Then we increased the length of the first 4-mer peptide by adding one residue at a time towards the N-terminus of TempL. Thus we synthesized five peptides from the central to the N-terminus of TempL having four, five, six, seven and eight residues (T-4mer, T-5mer, T-6mer, T-7mer and T-8mer) respectively (Table 1). It can be observed that among these five peptides T-4mer, T-5mer and T-8mer contain aromatic residues at both ends (Table 1). Though T-6mer and T-7mer possess an aromatic residue, namely, phenylalanine, only at their C-termini, an aromatic residue is positioned not far from the N-termini of these two peptides too. For example, in the T-6mer and T-7mer peptides, the second and third residue respectively from their N-termini is also an aromatic residue, namely, tryptophan (Table 1).

**Table 1:** Sequences and molecular masses of Temporin L-derived sequences

Peptide name	Peptide sequence^a^	Molecular weight (g/mol)	
		Calculated	Observed^b^
Temporin L (TempL)	F V Q W F S K F L G R I L	1639.9	1639.8
T-4mer	F S K F	526.63	527.2
T-5mer	W F S K F	712.84	713.3
T-6mer	Q W F S K F	840.97	841.3
T-7mer	V Q W F S K F	940.1	940.4
T-8mer	F V Q W F S K F	1087.27	1087.5
T-8merF1A	**A** V Q W F S K F	1011.18	1011.4
T-8merV2A	F **A** Q W F S K F	1059.22	1059.6
T-8merQ3A	F V **A** W F S K F	1030.22	1030.4
T-8merW4A	F V Q **A** F S K F	972.14	972.4
T-8merF5A	F V Q W **A** S K F	1011.18	1011.5
T-8merS6A	F V Q W F **A** K F	1071.27	1071.4
T-8merK7A	F V Q W F S **A** F	1030.18	1030.5
T-8merF8A	F V Q W F S K **A**	1011.18	1011.4
T-8mer-F1,W4,F5,F8A	**A** V Q **A A** S K **A**	743.85	744.3
T-8mer-V2,Q3,S6,K7A	F **A A** W F A A F	929.07	929.4
T-8mer-V2A,K7R	F **A** Q W F S R F	1087.23	1087.5
T-Cter-5mer	L G R I L	569.74	570.4

a The C-termini of the peptides are amidated (-CONH_2_).

b Observed mass of the peptides were determined by MALDI-TOF. Alanine replacement in T-8mer is indicated by bold letter.

All these five peptides were purified and characterized. As control peptides, the full length peptide, TempL, and the C-terminus peptide having the 9th to 13th amino acid residues of TempL with no aromatic residue were also synthesized and characterized. Besides, eight single-alanine substituted analogs of T-8mer peptide were also synthesized (Table 1). Molecular masses of all the synthesized peptides were confirmed (Table 1) by recording their MALDI-TOF mass spectra (Fig. S1). All the peptides studied were found to have >95% purity (Fig. S2), as confirmed by RP-HPLC by utilizing an analytical Waters XBridge peptide BEH C18 HPLC column (300 Å, 5.0 μm, 4.6 mm × 250 mm).

### Nonhemolytic and noncytotoxic nature of TempL-derived peptides

The hemolytic activity of all these peptides was examined against human red blood cells (hRBCs) (Fig. S3*A*). All the TempL-derived peptides showed negligible lyses of hRBCs at a concentration as high as 200 μM, whereas TempL showed >90% lyses of hRBCs at ∼40 μM concentration (Fig. S3*A*). Cytotoxicity of the peptides was tested against HepG2 cells by 3-(4,5-dimethylthizol-2-yl)-2,5-diphenyltetrazolium bromide (MTT) assay (Fig. S3*B*). Results showed 100% viability of HepG2 cells, treated with different TempL-derived peptides at 200 μM concentration. Overall, the results suggested the nonhemolytic/nontoxic nature of these TempL-derived peptides.

### TempL-derived peptides showed the circular dichroism (CD) spectra of collagen-like triple-helical signature

**Figure 1:**
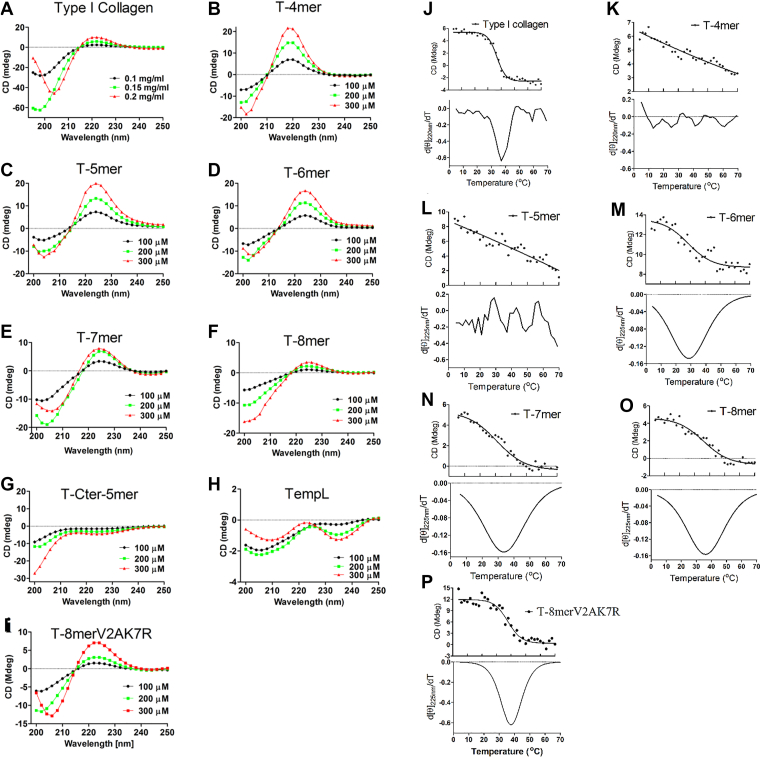
**Circular Dichroism study.***A* and *I*, CD spectra for secondary structure determination: *A*, rat tail type-I Collagen, (*B*) T-4mer, (*C*) T-5mer, (*D*) T-6mer, (*E*) T-7mer, (*F*) T-8mer, (*G*) T-Cter-5mer, (*H*) TempL and (*I*) T-8mer-V2A,K7R at different concentrations in Milli-Q water. *J* and *P*, unfolding melting studies: (*J*) rat tail type-I collagen, (*K*) T-4mer, (*L*), T-5mer, (*M*) T-6mer, (*N*) T-7mer, (*O*) T-8mer and (*P*) T-8mer-V2A,K7R. The concentration of type I collagen and TempL derived peptides was 0.2 mg/ml and 300 μM, respectively. Temperature was increased from 4 °C to 70 °C at an interval of 2 °C. Lower panels in each figure showing the first derivative of unfolding melting curves. TempL, temporin L.

Secondary structures of TempL-derived peptides were determined by recording their circular dichroism (CD) spectra at different concentrations in Milli-Q (MQ) water. Further, rat tail type-I collagen [stock concentration 2.5 mg/ml (w/v)] was diluted in MQ water [0.1, 0.15 and 0.2 mg/ml (w/v)] for recording its CD spectra. T-4mer, T-5mer, T-6mer, T-7mer and T-8mer showed positive peak at 220 to 225 nm and a cross over near 213 nm, corresponding to the PPII helix structure (Fig. 1) (14, 15, 16). Particularly, CD spectra of T-6mer, T-7mer and T-8mer peptides resemble the CD spectral signature of triple helical collagen for example rat tail type-I collagen (Fig. 1*A*) or collagen-mimetic peptides with positive peak at near 225 nm and negative peak at 200 to 210 nm (13, 17, 18, 19). However, the CD spectra of the C-terminus derived 5-mer peptide (T-Cter-5mer, Fig. 1*G*) and the parent peptide, TempL (Fig. 1*H*) did not show any positive ellipticity, characteristic of PPII helix in MQ water. Overall, the CD spectra of these peptides derived from the middle of TempL to its N-terminus revealed remarkable findings on the identification of short peptides that showed CD spectra of collagen-like triple-helical signature (20, 21).

### Melting curves of TempL-derived T-6mer, T-7mer and T-8mer showed sigmoidal nature with T-8mer exhibiting the highest melting temperature

Melting curves of triple helical collagen and collagen-mimetic compounds have been routinely employed for their characterization (13, 17, 22, 23). Melting temperature of each of the currently synthesized peptides was determined by recording the CD values at the respective wavelength in which it showed the maximum positive ellipticity, as a function of temperature. The first derivative of the mean residue ellipticity values of a peptide at various temperatures at a particular wavelength was used to find out its melting temperature (Fig. 1, *J*–*P*). Thermal melting curves of T-4mer and T-5mer showed linear nature indicating weaker self-assembly properties of these peptides. However, the melting curves of T-6mer, T-7mer and T-8mer showed appreciable sigmoidal nature with melting temperatures of ∼28 °C, 34 °C and 36 °C, respectively.

The sigmoidal nature of melting curve is characteristic of cooperative denaturation of collagen and collagen-mimetic triple-helical peptides with higher order supramolecular structures (17, 23, 24, 25). It is evident that the melting curves of T-6mer, T-7mer and T-8mer peptides appreciably mimic the pattern of melting curve of rat tail type-I collagen (Fig. 1*J*). Thus the results suggested that these TempL-derived peptides possess higher order structures that are found in collagen and collagen-mimetic peptides (17, 23, 24, 25).

### Characterization of self-assembly properties of single alanine-substituted analogs of T-8mer peptide

As per the results in the previous section, T-8mer peptide, showed the highest melting temperature among these TempL-derived peptides. Therefore, for further investigating the role of each of its amino acid residues in its folding, eight single alanine-substituted analogs of T-8mer peptide were synthesized after substituting its each amino acid residue with an alanine residue at a time (Table 1). The CD spectra of these single alanine-substituted T-8mer analogs were recorded (Fig. S4) for investigating their self-assembly and folding properties in aqueous environment. The maximum changes in its CD spectrum were observed when the aromatic tryptophan residue at the fourth position was replaced with an alanine residue. The positive peak of T-8mer at 225 nm, characteristic of its collagen-like triple-helical structure, moved to ∼220 nm as a result of tryptophan to alanine substitution and T-8merW4A showed an almost linear melting curve instead of a sigmoidal one. The T-8mer variant, T-8merF5A, with the aromatic phenylalanine residue at the fifth position substituted with an alanine residue also showed its positive peak shifted to a shorter wavelength from 225 nm. However, the two other variants, T-8merF1A and T-8merF8A with replacement of the corresponding aromatic residue by an alanine residue, exhibited relatively smaller shifts in their positive peaks towards the shorter wavelengths. Substitutions of aliphatic amino acid residues of T-8mer with alanine residues also demonstrated significant alterations in its CD spectra and the melting curve. For example, CD spectrum of T-8merQ3A showesd a drastic reduction in the ellipticity values along with shift of its positive peak towards a shorter wavelength. Due to a large reduction in the intensity of its positive peak, it was not possible to record the melting curve of T-8merQ3A (Fig. S4). T-8merS6A also exhibited its positive peak at a shorter wavelength than 225 nm and its melting curve deviated from sigmoidal to linear nature. Overall, most of the aromatic and aliphatic amino-acid residues seem to play crucial roles in the folding of T-8mer.

Two more analogs of T-8mer were synthesized. In one of these two analogs (T-8mer-F1,W4,F5,F8A), all four aromatic residues were substituted with four alanine residues leaving the four aliphatic residues intact. While in the other analog (T-8mer-V2,Q3,S6,K7A), all four aliphatic residues of T-8mer were replaced with four alanine residues and the four aromatic residues remained intact (Table 1). CD spectra of these two peptides (Fig. S4, *A* and *B*) indicated that more significant alterations occurred for the T-8mer analog (T-8mer-F1,W4,F5,F8A) in which all four aromatic residues were replaced with alanine residues while the aliphatic-residues remained unchanged. The melting curve of this analog (T-8mer-F1,W4,F5,F8A) could not be recorded while the melting curve of T-8mer-V2,Q3,S6,K7A was recorded (Fig. S4*D*). The stronger abrogative effect of the substitutions of multiple aromatic residues of T-8mer with alanine residues could be a reflection of the synergistic effect of the presence of two or more aromatic residues in its folding by participating in a network of π-π interaction.

### Design of a T-8mer variant, T-8mer-V2A,K7R which exhibited collagen-mimetic self-assembly property, similar to T-8mer

We aimed for a designer T-8mer-variant with collagen-mimetic properties. Considering the CD spectra and melting curves of the single alanine-substituted analogs of T-8mer peptide (Fig. S4, *A*–*D*), it appeared that the substitutions of valine and lysine residues respectively at the second and seventh positions with alanine residues manifested relatively lesser effects on its CD spectrum of collagen-like triple-helical signature and its melting temperature (Fig. S4). Therefore, for designing a new T-8mer variant which could maintain its collagen-mimetic properties, its valine and lysine residues at the second and seventh positions were substituted with alanine and arginine residues respectively and its structural and biological properties were investigated. Interestingly, this T-8mer variant, T-8mer-V2A,K7R like its parent peptide showed the CD spectrum with positive peak at ∼ 225 nm, characteristic of collagen-like triple helical structure (Fig. 1*I*). Remarkably, the melting curve of T-8mer-V2A,K7R showed significant sigmoidal nature with the melting temperature of ∼ 39 deg C (Fig. 1*P*). Overall, the new T-8mer variant, T-8mer-V2A,K7R appreciably retained the collagen-mimetic triple-helical signature and self-assembly properties of the identified TempL-derived, collagen-mimetic peptide, T-8mer.

### Characterization of nano-fibrillar structure of TempL derived peptides, T-6mer, T-7mer, T-8mer and T-8mer variant, T-8mer-V2A,K7R

**Figure 2:**
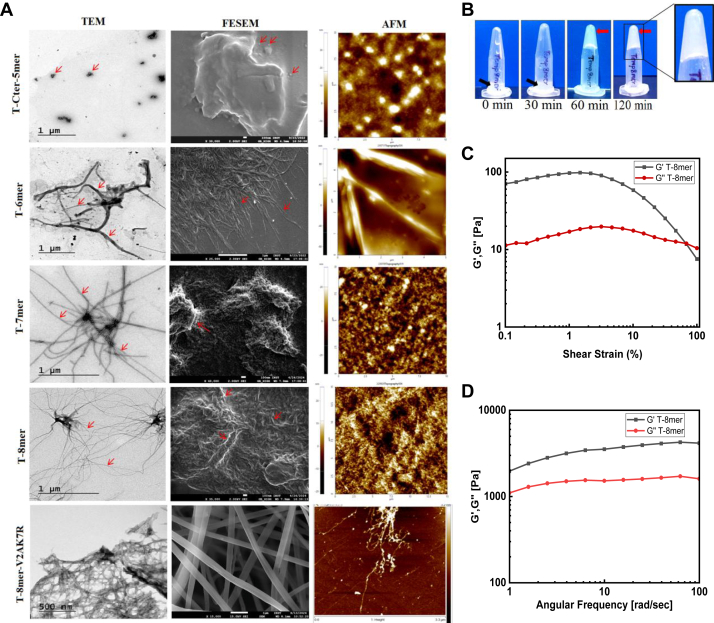
**Nanostructures formation studies.***A*, representative TEM (5 mg/ml, w/v in MQ water), field-emission scanning electron microscopic (1 mg/ml, w/v in MQ water) and atomic force microscopic (3 mg/ml, w/v in MQ water) micrographs of temporin L derived peptides. The concentration of each peptide was 5 mg/ml (w/v) in MQ water (pH 7.4). *B*, hydrogel formation study. Inverted tube test for visual hydrogel formation by T-8mer at different time points. T-8mer peptide was dissolved at a concentration of 1.5% (w/v) in MQ water (pH 7.4). Rheology of the T-8mer peptide. *C*, strain sweep at 1.0% by weight peptide concentration in MQ water at a temperature of 37 °C and a frequency of 10 rad s^-1^ shown as G′ and G′′. *D*, frequency sweep at 1.0% by weight peptide concentration in at a temperature of 37 °C and 1% strain shown as G′ and G′′.

In order to understand the microstructural properties of different TempL-derived self-assembling peptides, transmission electron microscopic (TEM) images of these peptides were collected. TEM images of TempL-derived peptides were collected following their negative staining to investigate their self-assembly properties and nanostructure (Fig. 2*A*). T-4mer and T-5mer showed beaded nanostructures with average diameter of approximately 30 nm (Fig. S5, Table 2) as determined from the TEM images. While T-6mer, T-7mer and T-8mer formed fibrillar nanostructures with width in the range of 10 to 16 nm and length in the range of 1.6 to 2.0 μm (Table 2). On the other hand, TempL and the peptide derived from the C-terminus of TempL, T-Cter-5mer, showed spherical aggregated structure (Figs. 2*A* and S5*C*). Overall, the results suggested that T-6mer, T-7mer and T-8mer formed prominent fibrillar nanostructures.

**Table 2:** Nanostructures size as determined from TEM images by using Image J software (https://imagej.net/ij/) (N = 30)

Peptide name	Morphology of nanostructure	Diameter of spheres/Width of nanofibers	Length of nanofibers
T-Cter-5mer	Aggregated spherical	-	-
T-4mer	Beaded nanostructure	∼29 nm	-
T-5mer	Beaded nanostructure	∼35 nm	-
T-6mer	Fibrillar	∼16 nm	∼2 μm
T-7mer	Fibrillar	∼10 nm	∼1.6 μm
T-8mer	Fibrillar	∼10 nm	∼1.7 μm
T-8mer-V2A,K7R	Fibrillar	∼13 nm	∼1.3 μm

Further, for examining the surface morphology of these self-assembling peptides, field-emission scanning electron microscopic (FESEM) images of these peptides were taken and shown next to the TEM images in Figure 2. FESEM images support the different microstructures of TempL-derived peptides, obtained from TEM studies. Besides, the surface morphology of these TempL-derived peptides were also visualized by collecting their atomic force microscopic (AFM) images as shown in the right side of the FESEM images of the peptides (Fig. 2). Overall, these microscopic studies fairly correlate with each other, reinforcing the identification of new self-assembling peptides with various nanostructures from the aromatic residue-rich, amino-terminus of TempL. Interestingly, the new designer variant of T-8mer peptide (T-8mer-V2A,K7R), also adopted nano-fibrillar structure with average width of 13 nm and length, 1.3 μm respectively (Table 2). TEM, FESEM and AFM images of T-8mer-V2A,K7R are shown in Figure 2 that are consistent with its nanofibrillar structure.

Dynamic Light Scattering experiments showed that TempL, T-4mer, and T-5mer exhibited multiple aggregated states, while T-6mer, T-7mer, T-8mer, and T-8mer-V2A,K7R demonstrated uniform nanostructure (Fig. S6) with a single aggregated state.

### TempL derived, T-8mer forms supramolecular hydrogel

The ability of these peptides to form supramolecular assembly in the form of hydrogel was also investigated. All the peptides, tested for hydrogel formation were dissolved in MQ water in an eppendorf tube at a concentration of 1.5% (w/v) and incubated at 25 °C in static condition. Hydrogel formation was determined by examining whether a sample flows or not when the eppendorf tube is inverted (26). Images of inverted tubes were acquired at different time points (Fig. 2*B*). T-8mer did not form hydrogel till 30 min and was present in the form of a viscous solution. However, it formed hydrogel after incubation of 60 min (Fig. 2*B*). As described, though, we have examined the time required to form a stable gel (Fig. 2*B*) for 1.5% (w/v) T-8mer peptide, its minimum gelation concentration (27), at which the solution of the peptide loses its fluidity and undergoes gelation, has not been assessed. However, T-4mer, T-5mer, T-6 mer and T-7mer did not form any hydrogel up to incubation period of 7 days and remained in the liquid state (Fig. S7*A*). Hydrogel formation indicated supramolecular assembly of PPII containing T-8mer, having cross-linked polymeric networks, saturated with water. It is notable that collagen mimetic, designer variant, T-8mer-V2A,K7R also formed hydrogel after 60 min incubation, however it melted as shown in Figure S7*B*.

### T-8mer hydrogel exhibited mechanical properties

The viscoelastic properties of T-8mer hydrogel were investigated by strain and frequency sweeps rheology methods. The strain/amplitude sweep analysis of T-8mer hydrogel (Fig. 2*C*) provided valuable insights into this peptide gel's mechanical properties at the condition of increase in strain. The crossover point of G′ and G″ (Fig. 2*C*) is indicative of the gel−sol transition and the linear viscoelastic region, as reported for rat tail collagen and collagen mimetic peptides (28). The storage modulus (G′) and loss modulus (G″) displayed frequency-independent behavior, with G′ predominating over G″ throughout the range as shown in Figure 2*D*, indicating the solid like behaviour of T-8mer hydrogel (29). Across the tested frequency range, the T-8mer hydrogel exhibited a consistent and stable response as shown in Figure 2*D*. These combined frequency and amplitude sweep analyses of T-8mer hydrogel (Fig. 2, *C* and *D*) provided data on the significant rheological properties of T-8mer hydrogel. Overall, TempL-derived, T-8mer, not only showed self-assembly properties, but it spontaneously formed supramolecular hydrogel with appreciable mechanical properties, as reported for rat tail collagen and collagen mimetic peptides (17, 30).

### TempL derived, T-6mer, T-7mer, T-8mer and T-8mer-V2A,K7R peptides showed collagen-mimetic, cell-adhesion property

**Figure 3:**
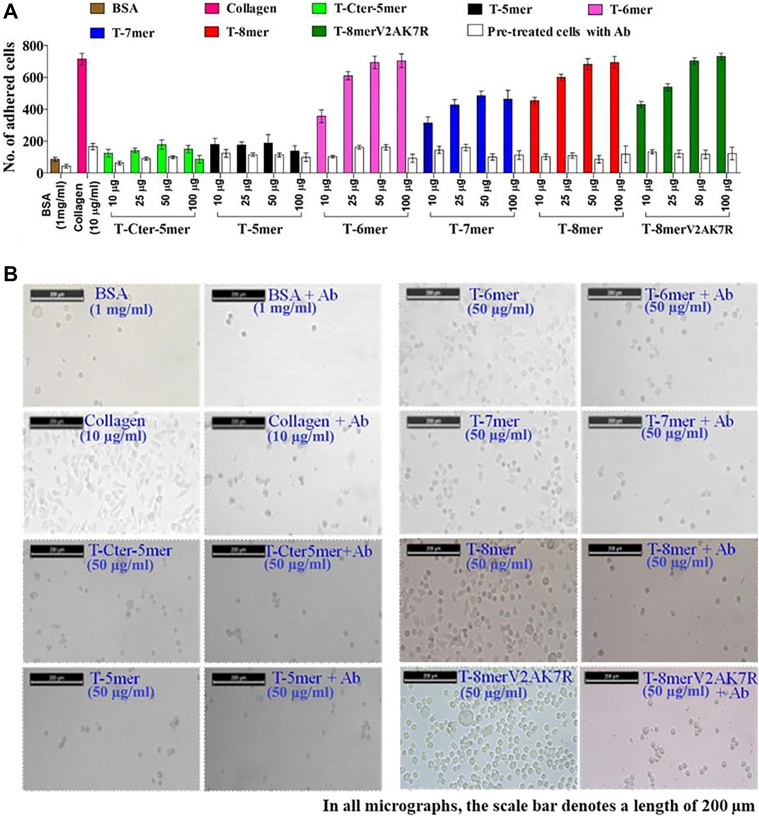
**Study of adhesion of HepG2 cells on the coatings of different TempL-derived peptides and other control molecules and examination of effects of collagen-receptor antibody treatment onto it.***A*, colored bars indicating the number of HepG2 cells adhered on heat-denatured BSA, Collagen, T-Cter-5mer, T-5mer, T-6mer, T-7mer and T-8mer and T-8mer-V2A,K7R coated surface at different concentration (10 μg/ml, 25 μg/ml, 50 μg/ml and 100 μg/ml) as shown on the X-axis. White clear bars indicated the number of anti-integrin α2β1 antibody pretreated HepG2 cells, adhered onto the surface of above mentioned molecules. Cell adhesion to heat denatured BSA and rat tail type-I collagen coated well was used as negative and positive control, respectively. Statistical analysis was performed to compare denatured BSA with collagen and all the tested peptides, by employing one-way analysis of variance using Tukey's multiple comparison tests. A *p* value of <0.05 was considered as significant. *B*, light microscopic images showing the anti-integrin Ab untreated and anti-integrin Ab pretreated adhered cells onto heat denatured BSA (1 mg/ml, w/v), collagen (10 μg/ml, w/v) and TempL derived peptide (50 μg/ml, w/v) coated surface. TempL, temporin L.

Collagen, the major extracellular matrix protein and the most abundant structural proteins in mammals is known for its cell adhesion property (31, 32, 33, 34). To investigate if any of the TempL-derived peptides possess any cell-adhesion property like collagen, the adhesion of HepG2 cells was examined onto the surfaces of these peptides. These cells have been utilized for similar purposes in earlier studies (33). HepG2 cells were cultured onto 96-well microplate coated with rat tail type-I collagen, heat denatured bovine serum albumin (BSA), T-Cter-5mer, T-5mer, T-6mer, T-7mer, T-8mer and T-8mer-V2A,K7R for 1 h at 25 °C and adhered cells were counted using light microscope (Figs. 3, *A* and *B*, and S8). Cell adhesion to rat tail type-I collagen and heat denatured BSA were used as the positive and negative control, respectively. Cell adhesion to the rat tail type-I collagen substrate was taken as 100% reference level.

Significant adhesion of HepG2 cells occurred onto the wells coated with rat tail type-I collagen, T-6mer, T-7mer and T-8mer peptides (Fig. 3*A*). Surface coated with T-Cter-5mer, showed only ∼20% adhesion of cells, similar to that observed onto the denatured BSA coated surface. Significant adhesions of HepG2 cells were observed onto T-8mer-variant, T-8mer-V2A,K7R-coated wells also. Of these peptides, T-6mer, T-8mer and T-8mer-V2A,K7R peptides exhibited comparable and high adhesions of cells (Fig. 3*A*). Results suggested that PPII containing peptides with increasing fibrillary structure promote cell adhesion as was reported in earlier studies (35, 36) also.

Integrin function as collagen receptors and various integrins comprising of α and β subunits are responsible for binding of the cells to extracellular collagen matrix (37, 38). We have investigated if collagen receptor, integrins, are involved in the adhesions of HepG2 cells onto the surface of different TempL-derived peptides including the T-8mer designer variant, T-8mer-V2A,K7R. HepG2 cells were pre-treated with integrin α2β1 antibody, and further adhesions of these cells were examined onto the surface of all these TempL-derived peptides as mentioned above and rat tail type I collagen which was used as the positive control. Results suggested that pre-treatment with integrin α2β1 antibody significantly compromised the adhesions of HepG2 cells onto the surface of these TempL-derived peptides as well as rat tail type I collagen protein (Figs. 3, *A* and *B*, and S8). Images of adhesions of HepG2 cells with or without antibody treatment onto the surface coated with 50 μg/ml concentration of different TempL-derived peptides are shown in Figure 3*B* whereas the same for the concentrations of 10 μg/ml, 25 μg/ml, and 100 μg/ml of different TempL-derived peptides are shown in Figure S8. The results could be implicated to a prominent role of collagen receptor, integrin in the adhesions of HepG2 cells onto the surface of TempL-derived peptides which further pointed out towards the collagen-mimetic property of these peptides.

### T-6mer, T-7mer, T-8mer and T-8mer-V2A,K7R peptides induced cytoskeletal organization in HepG2 cells like collagen

**Figure 4:**
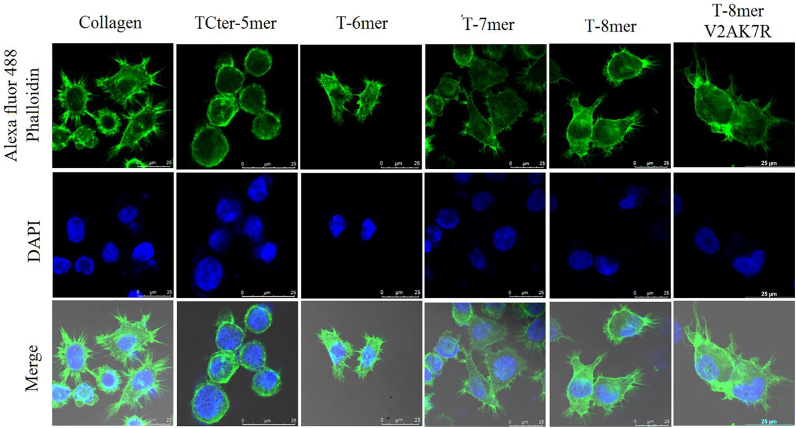
**Cytoskeletal organization of HepG2 cells as a function of substrates: rat tail type-I collagen, T-Cter-5mer, T-6mer, T-7mer and T-8mer (the concentration of collagen and peptide was 10 μg/ml and 50 μg/ml, respectively).** Cells were fixed and stained for actin stressfibers (Alexa fluor 488–phalloidin; *green*) and nuclei (DAPI; *blue*) after 3 h of adhesion in serum-free medium and imaged using a confocal laser scanning microscope (60× magnification).

To get further insight on the adhesions of HepG2 cells, cytoskeletal organization of adhered HepG2 cells onto the surfaces of different TempL-derived peptides and type-I Collagen was examined by the staining of actin stress fibers with Alexa fluor 488-Phalloidin, green and nuclei with DAPI, blue of these cells as a function of substrate by confocal microscopy. HepG2 cells were seeded onto type- I Collagen, T-Cter5mer, T-6mer, T-7mer, T-8mer and T-8mer-V2A,K7R coated chamber slides. HepG2 cells displayed well developed actin cytoskeletal structure on rat tail type-I collagen, T-6mer, T-7mer, T-8mer and T-8mer-V2A,K7R coated surfaces (Fig. 4). Additionally, adhesion of HepG2 cells and spreading of their actin cytoskeletal network were extensive onto the surface coated with T-6mer, T-7mer, T-8mer and T-8mer-V2A,K7R like that onto the surface of rat tail type-I collagen, suggesting collagen-mimetic biological properties of these peptides. In contrast, the actin cytoskeletal organization of HepG2 cells was much less pronounced onto the surface of non-PPII helix peptide, T-Cter-5mer, and most of these cells remained spherical, as expected (Fig. 4). Overall, TempL-derived, T-6mer, T-7mer, T-8mer and T-8mer-V2A,K7R peptides with PPII structure showed the biological properties of collagen, including the adhesion and cytoskeletal organization (33, 39) of HepG2 and therefore these peptides could be treated as new collagen-mimetic peptide biomaterials.

## Discussion

The results presented here demonstrate the identification and characterization of three self-assembling collagen-mimetic peptides (T-6mer, T-7mer and T-8mer) from the aromatic amino acid-rich region of a frog-origin peptide, TempL (Figure 1, Figure 2, Figure 3, Figure 4; Tables 1 and 2). Further, to provide data on understanding the proof of concept about the primary structure of the most active peptide (T-8mer) among these TempL-derived peptides, we have designed its variant. This variant (T-8mer-V2A,K7R) also demonstrated structural and biological properties of its parent collagen-mimetic peptide. Remarkably, none of these TempL-derived, collagen-mimetic peptides possesses the four abundant residues that are found in collagens, namely, glycine, proline, alanine and hydroxyproline suggesting that the presently discovered peptide scaffolds are novel and comprised of new combinations of amino acid residues. Of the five synthesized peptides from the amino terminus of TempL, 4-residue (T-4mer) and 5-residue (T-5mer) exhibited CD spectra (peak at 220–222 nm) of PPII helix structure (Figure 1), almost linear melting curve (Figure 1) and nano-spherical assembly (Fig. S5, Table 2). However, the 6 to 8 residue peptides, namely T-6mer, T-7mer and T-8mer peptides showed CD spectra of PPII triple-helical structure (peak at ∼225 nm) like collagen protein or collagen-mimetic peptides.

It is to be mentioned that the CD spectra of PPII helix and collagen triple-helix show similarities since collagen is comprised of three intertwined PPII helices. While PPII helix is single-stranded, self-assembly of three PPII helices creates triple-helical collagen structure. Thermal denaturation studies can reveal the differences in their self-assembly properties and thus definitively distinguish between triple-helical collagen and single-stranded PPII helix (40). Whereas thermal denaturation of collagen triple helices is a cooperative process which is reflected in its sigmoidal loss of ellipticity values, the same event for single stranded PPII helix shows linear reduction in ellipticity values with no cooperative transition.

In the current investigation, along with collagen-mimetic triple-helical structure, T-6mer, T-7mer and T-8mer peptides also showed sigmoidal melting curves with melting temperatures between 28 to 36 °C. Further, these peptides also demonstrated nanofibrillar assembly as evidenced by TEM, FESEM and AFM studies (Figure 2) with fiber length of ∼ 2 μm (Table 2). Similar sizes of fiber length have been reported for other collagen-mimetic peptides also (13, 41). Moreover, self-assembly properties of these TempL-derived peptides were supported by dynamic light scattering studies with these peptides (Fig. S6). T-8mer showed the highest melting temperature of 36 °C among these peptides. Interestingly, the 8-mer peptide, T-8mer at 1.5% (w/v) readily formed hydrogel (Fig. 2*B*) indicating its ability to adopt higher order three dimensional structure with absorption of water (Fig. 2*B*). Further, rheological studies indicated that the hydrogel of T-8mer peptide possesses significant mechanical properties (Fig. 2, *C* and *D*). To form hydrogel with mechanical properties for a short peptide with only eight amino acid residues suggests that the identified T-8mer peptide possesses an interesting combination of amino acid residues which confers it a significant self-assembly property. It is fascinating that T-8mer, having just eight amino acid residues, formed hydrogel with appreciable mechanical properties. However, for considering its potential biomedical application, its stability under physiological conditions and long-term structural maintenance (>24–48 h) need to be examined that have not been studied in the current investigation. Not only that T-6mer, T-7mer and T-8mer peptides showed the CD spectra of collagen-like triple-helical signature and sigmoidal nature of melting curves but also these peptides demonstrated the biological property of collagen or collagen-mimetic peptides. Evidently, these three peptides demonstrated adhesion of HepG2 cells onto the coating of their surfaces like collagen for example, rat tail type-I collagen which was employed as a positive control (Figs. 3 and S8). Remarkably, when these cells were pre-treated with antibody of collagen receptor namely, integrin α2β1 antibody, adhesion of HepG2 cells reduced drastically onto the surfaces of TempL-derived, T-6mer, T-7mer and T-8mer peptides (Figs. 3 and S8). Similar reduction in cell-adhesion was also observed when rat tail type-I collagen was used instead of these TempL-derived peptides. The results suggested a possible role of collagen-receptor in the adhesion of HepG2 cells onto the surfaces of these TempL-derived peptides. In continuation with the cell-adhesion property of T-6mer, T-7mer and T-8mer peptides, cytoskeletal organization was observed in HepG2 cells following their adhesions onto the surface of these three TempL-derived peptides (Fig. 4) which corroborated with their collagen mimetic nature. Since HepG2 cells express different collagen-binding receptors, these cells have been used as suitable model for studying cell-matrix interactions and investigating the ability of peptides to mimic collagen by examining cell adhesion and actin stress fiber development as in the current study. However, how these newly identified TempL-derived collagen-mimetic peptides behave in other cell types including fibroblasts, for example NIH 3T3 and mesenchymal stem cells that are often employed in extracellular matrix research remains to be seen. Taking clues from the results on the effects of single alanine-substitutions for each amino acid residue of T-8mer peptide, we envisaged to design its variant which would retain the collagen-mimetic self-assembly and cell-adhesion properties of its parent peptide. Our results suggested on the successful design of T-8mer variant, T-8mer-V2A,K7R which showed CD spectra of collagen-like triple-helical signature, sigmoidal melting curve with slightly elevated melting temperature (∼39 °C) than its parent peptide, nanofibrillar self-assembly and collagen-receptor sensitive cell-adhesion property (Figure 1, Figure 2, Figure 3, Figure 4, Tables 1 and 2). Moreover, T-8mer-V2A,K7R also formed hydrogel like T-8mer but it showed weaker stability (Fig. S7*B*). These results suggested that majority of the individual amino acid residues of T-8mer peptide are vital for maintaining its collagen-mimetic structure and self-assembly properties and in T-8mer-V2A,K7R, we found the right alternative combination of amino acid residues for designing its new collagen-mimetic variant.

However, the peptide derived from the C-terminus of TempL, T-Cter-5mer, having no aromatic residue did not show any CD spectrum of collagen-like triple-helical signature and also exhibited insignificant cell adhesion property (Figure 1, Figure 2, Figure 3, Figure 4, Tables 1 and 2).

Though aromatic residues are mostly uncommon in collagen, the presence of phenylalanine residues at the two ends of glycine, proline and hydroxyproline containing ∼30-mer model collagen peptides have been shown to promote self-assembly of these triple-helical peptides to higher order structures (13, 42). In the current investigation, TempL-derived, T-8mer peptide showed the maximum melting temperature, formed hydrogel and exhibited the highest cell-adhesion properties among these TempL-derived peptides. The results could be implicated for a possible contribution of the aromatic residues in the head and tail of the peptide in terms of participating in the head to tail aromatic stacking interactions among the individual triple helices in its supramolecular self-assembly. In other two TempL-derived, collagen-mimetic peptides, namely T-6mer and T-7mer besides having aromatic phenylalanine residues at their carboxy-termini also possess aromatic residues close to their amino termini (Table 1).

It is to be mentioned that a) the identified peptides possess eight residues maximum (T-8mer) as compared to collagens that possess more than 1400 residues; moreover, the designed collagen-mimetic peptides reported in the literature are around 30-residue long to our knowledge; b) despite having a small number of amino acid residues, (T-8mer, eight residues; T-7mer, seven residues and T-6mer, six residues), these peptides exhibited the usual steps for forming collagen-like assembly, namely, self-assembly of peptide chains to adopt collagen-mimetic triple-helical structure and formation of nanofiber assembly; c) additionally, T-8mer formed three dimensional water swollen polymeric network of hydrogel, indicating its ability to show further higher order structure.

## Conclusion

Taken together, the current investigation demonstrated the identification of novel collagen mimetic peptide scaffolds from the N-terminus of a frog AMP, TempL. To our knowledge this is the first report on the discovery of collagen-mimetic peptides from a naturally occurring peptide whose property related to collagen has not been reported. The results also show the discovery of a very short peptide (T-8 mer) which forms hydrogel and show collagen-mimetic properties and thus open up new avenues related to the design of new collagen mimetic peptides on this aromatic-residue rich scaffold and exploring their structural and biological properties and finding biomedical application of such peptide-scaffolds. In this context, it is to be mentioned that all these TempL-derived peptides exhibited desired safety parameters, showing totally nonhemolytic and non-cytotoxic nature (Fig. S3) till 200 μM concentration which adds values for considering further studies in exploring the potential of these peptides. The current results further strengthen a notion that investigation on a judicially selected small segment of a peptide could lead to the identification of new structural scaffolds and bioactive molecules.

## Experimental procedures

### Reagents and fluorescent probes

Rink amide 4-methylbenzhydrylamine resin (loading capacity: 0.36–0.78 mmol/g), N-α- fluorenylmethoxycarbonyl (Fmoc) and necessary side-chain protected amino acids were purchased from Novabiochem. Coupling reagents for peptide synthesis including Oxyma Pure [ethyl 2-cyano-2-(hydroxyimino) acetate] (8.51086) and O-(6-chlorobenzotriazol-1-yl)-N,N,N′,N′-tetramethyluronium hexafluoro-phosphate (HCTU) (8.51012) were purchased from Novabiochem, N,N′-diisopropylcarbodiimide (38,370), N,N-diisopropylethylamine (DIPEA)and NMM (N-methylmorpholine) were purchased from Sigma. Dichloromethane, N,N-dimethylformamide (DMF), piperidine, diethyl ether and trifluoroacetic acid (TFA) were of standard grades and procured from reputed local companies. Triton X-100 (T8787), Ninhydrin (N4876) and KCN (60,178), p-cresol (C7525), thioanisole (88,470), 1,2-ethanedithiol (02,390 Ammonium persulfate (A3678, N,N′-methylbisacrylamide, Acylamide, and rat tail type-I collagen (C3867) were purchased from Sigma. For cell culture, RPMI 1640, Fetal Bovine Serum, 100X Antibiotic–antimycotic and 0.25% Trypsin-EDTA (1X) were purchased from Gibco/Invitrogen. Alexa fluor-488-phalloidin (A12379) and Hoechst 33,342 (62,249) were purchased from Thermo Fisher Scientific. Sterile T-25 cm^2^ and T-75 cm^2^ polystyrene tissue culture flasks, 96-well plates (3599) and 6-well plates (3506) were from Corning Incorporated.

### Cell line

Human hepatoma cell line, HepG2 was obtained from CSIR-Central Drug Research Institute (CSIR-CDRI), Lucknow cell line repository. The cell lines were maintained by the usual protocol in an Innova CO-170 CO_2_ incubator.

### Synthesis of peptides

All the peptides were synthesized manually and with a peptide synthesizer (PS3 model, Protein Technologies, Inc.) using 9- Fmoc solid phase method on rink amide 4-methylbenzhydrylamine resin (43, 44). Fmoc strategy is based on the orthogonal concept that the two protecting groups belong to independent chemical classes and are removed by different mechanisms. Initially, 100 mg of resin was taken in a glass vessel. For loading the first amino acid from the C-terminal of the peptide sequence on the resin, the Fmoc protecting group of the resin was removed. Removal of the Fmoc group is achieved usually with 30% piperidine in DMF and deprotection was confirmed by positive Kaiser Test (45), which gives blue color on reacting with free α-amino group. Chloranil test was performed for checking the deprotection of imino acids like proline which gives blue color at room temperature with free imino groups and yellow (or colorless) with bound imino groups. Before coupling, the resin was washed several times to remove all the piperidine. Afterwards, in case of manual peptide synthesis, the Fmoc protected first amino acid (2.5-fold of the loading capacity of the resin), Oxyma and HCTU dissolved in DMF was added into the glass vessel containing resin and then desired amount of DIPEA was added to it (*in situ* activation). Coupling reaction was checked after 3 hours by negative Kaiser Test, which gives yellow color (or colorless) due to the absence of free amino group. In case of peptide synthesizer, Fmoc protected amino acid and HCTU dissolved in 0.4 M NMM (in DMF), then added into a glass vessel. Coupling reaction was checked after 1 hour. Further coupling reaction was carried out by employing Oxyma/ N,N′-diisopropylcarbodiimide or Oxyma/HCTU/DIPEA or HCTU/NMM reagents. Subsequently, rests of the amino acids in the peptide sequence from C- to N-terminal were coupled to the existing chain one after another. In case of incomplete deprotection or coupling reaction, the process was repeated. After the synthesis was over, each peptide was cleaved from the resin with simultaneous deprotection of side chains by treatment with a mixture of TFA-phenol-thioanisole-1,2-ethanedithiol -H_2_O (82.5:5:5:2.5:5 v/v) (reagent K) for 6 to 7 h and precipitated with dry ether (43).

### Purification of peptides with RP-HPLC

All of the peptides were purified by RP-HPLC on a semi-preparative Waters C18 column using a linear gradient of 10 to 90% acetonitrile/water in 40 min with a flow rate of 2.0 ml/min. Both acetonitrile and water contained 0.1% TFA. Molecular weight of purified peptides was confirmed by MALDI-TOF mass spectra analysis. All the purified peptides were freezed in liquid nitrogen and lyophilized to remove the solvent and get the peptides in powder form. Purity of peptides was checked by RP-HPLC on a analytical Waters XBridge peptide BEH C18 HPLC column (300 Å, 5.0 μm, 4.6 mm × 250 mm).

### Haemolytic activity assay of the peptides

Haemolytic activities of the peptides were performed as reported previously (10, 46). Briefly, fresh human red blood cells (hRBCs) were collected in the presence of an anticoagulant from a healthy volunteer for whom we have an approval from the Institutional Ethics Committee (No. CDRI/IEC/2019/A1). Further, the studies abide by the Declaration of Helsinki principles, and all the subjects were given written informed consent. Fresh hRBCs were washed with PBS (2–3 times) until the supernatant was clear. Hemolytic activity of the peptides was examined against 4% of the hRBCs in PBS by assaying the ability of the peptides to lyse the hRBCs. The peptide solution (100 μl) in PBS at various concentrations were placed into microcetrifuge tubes and mixed with 100 μl of the 8% hRBCs suspension. These mixtures were then incubated at 37 °C for 45 min in a shaking water bath to allow for the hemolysis to take place. After 45 min mixers were centrifuged at 5000 rpm for 5 min. Aliquots (100 μl) of the supernatant were transferred into a 96-well culture plate, and the hemoglobin release was measured by checking the UV absorbance of the samples at 540 nm using a microplate reader (BIO-TEK). In this assay: as a negative control, untreated red blood cell suspension was taken and 5% Triton X-100 containing solution of red blood cells was used as the positive control. Each assay was repeated for 3 times. Percentage of hemolysis was calculated as follows:

$$\%\;\text{Haemolysis}=\left[\left(A_{\text{sample}}\;–\;A_{\text{blank}}\right)/\;\left(A_{\text{triton}}\;–\;A_{\text{blank}}\right)\right]\times 100$$

### Cell viability assay

To examine the cytotoxic effects of the peptides, HepG2 cells were cultured in Dulbecco’s modified Eagle’s medium (DMEM) supplemented with 1% Anti-Anti-100X (Antibiotic-Antimycotic) and 10% fetal bovine serum at 37 °C in a humidified chamber under a 5% CO_2_ atmosphere. Growth inhibition was evaluated using MTT assays to assess cell viability as described earlier (47, 48). Cells were seeded at a density of 5 × 10^4^ cells/well into a 96-well culture plate and incubated at 37 °C for 24 h. After incubation different concentrations of each peptide in DMEM were added to the wells, and the cells were incubated for an additional 24 h at 37 °C. Thereafter, 10 μl of MTT (5 mg/ml) was added to each well, and the plate was incubated for 4 h. The supernatants were then removed, and 50 μl of DMSO was added to each well to dissolve any remaining precipitate. Finally, the absorbance at 550 nm was measured using an ELISA reader. The viability of the peptide-treated cells was calculated with respect to the control cells of 100% viability.

### Recording of CD spectra of the peptides by spectropolarimeter and analysis

To investigate the conformation of peptides, CD spectra of the peptides were recorded in JASCO J-1500 spectropolarimeter equipped with temperature controller. The spectrometer was calibrated routinely with 10-camphorsulphonic acid. The samples were scanned at 20 °C in Milli-Q water with the help of capped quartz cuvette of 0.2 cm path length at a continuous wavelength range of 250–200 nm. An average of three scans was taken for each sample with a scan speed of 50 nm/min and data pitch of 1 nm. However, for clear visualization of spectra, data pitch of 2 nm was used to plot the graph.

To investigate thermal denaturation, CD spectra of peptides were recorded in Milli-Q water by measuring mean residual ellipticity at their respective maximum positive ellipticity wavelength ranging between 220 to 225 nm, while temperature was varied from 4 °C to 70 °C at a rate of 0.2 °C/min (24, 49, 50).

### Transmission electron microscopic (TEM) study

Peptides were dissolved in MQ water (5 mg/ml, w/v) and left it for half an hour at the room temperature to allow the peptide to aggregate. 10 μl of this peptide solution was poured onto a freshly glow discharged carbon-coated copper grid for adsorption. The sample was left for 5 min and the excess solution was adsorbed by using clean filter paper. The grid was negatively stained with aqueous uranyl acetate for 30 s. Grids were air-dried before being examined and observed under a Jeol JEM1400 TEM at 80 kV. The images of a peptide sample were analyzed by using a GatanOrius 2KX2K CCD camera.

### Field emission scanning electron microscopy study

The morphological attributes and surface topography of the peptides were evaluated employing a Field Emission Scanning Electron Microscope (JEOL JSM-7610FPLUS) operating at 15 kV. The peptide solutions, with a concentration of 1 mg/ml, were formulated in ultra-pure Milli-Q water and drop casted onto silicon wafers. The specimens were allowed to stabilize at room temperature overnight. Subsequently, desiccation of the samples occurred within a vacuum-sealed desiccator. The samples were sputter-coated with gold for 80 s prior to imaging (28).

### Atomic force microscopic (AFM) analysis

The morphology of the self-assembled structures of all five peptides was analysed using Park XE70 Atomic Force Microscope. The peptide samples were dissolved in HPLC purified water at a concentration of 3 mg/ml and incubated at a temperature of 37 °C for duration of 4 days 20 μl of each sample solution was vigorously mixed and then applied onto freshly cleaved mica foils using a drop-casting method. The peptides were evenly spread across the surface using a spin coater. The samples were dehydrated for an extended period of time in a vacuum environment. The following day, the surface topography of each sample was examined using the tapping mode of AFM. Images were analyzed and processed with Park XEI image processing software (https://www.manualslib.com/manual/683128/Park-Systems-Xe-70.html) (51, 52).

### Dynamic light scattering study of self-assembling peptides

Size distributions of nanostructures formed by the peptides were measured by using a Malvern ZetasizerNano ZS (Zetasizer Ver. 7.11, Malvern Instruments; https://www.malvernpanalytical.com/en/support/product-support/software/zetasizer-family-software-update-v7-11) with a backscattering detection at 173°, equipped with a He−Ne laser (l = 632.8 nm) and dispersant RI = 1.33. All the peptides were diluted to 0.01% wt/vol, followed by standing at room temperature for 1 h before measurements. For each sample, 1 ml of the peptide solution was added into a clear disposable polystyrene cell and each sample was equilibrated at 25 °C for 2 min prior to measurement. Three measurements for each sample were performed, and the final result was calculated as the average of these measurements (53, 54).

### Hydrogel formation study

Peptides were dissolved in MQ water (pH 7.4) at a concentration of 1.5% (wt/vol) and incubated at 25 °C in static condition. The appendorf tubes containing peptide solution were inverted to check whether solution flows down or not. Images were acquired at different time intervals (17).

### Rheology

The investigation of the linear viscoelastic region, storage modulus, and loss modulus was conducted using an MCR (Modular Compact Rheometer) 302 advanced Rheometer apparatus employing parallel plate (PP-25) geometry at a physiological temperature of 37 °C. The gel specimen was prepared 1 day prior to the rheological property assessment. Subsequently, the hydrogel was placed between the plates, and the spindle was lowered to establish a 0.2 mm gap for measurement. Amplitude sweep analyses were performed over a range of 0.1 to 100% strain at 10 rad/s to identify the crossover point of storage (G′) and loss (G″) moduli, indicative of the gel−sol transition in the hydrogel. The linear viscoelastic range was determined through amplitude sweep studies, and the corresponding amplitude was utilized in subsequent frequency sweep experiments. The frequency sweep oscillatory rheology was executed from 0.1 to 100 rad/s at 1% strain within the linear viscoelastic region to illustrate the stability of the gel (29).

### Cell adhesion assay

Corning costar 96-well microwell plates were coated with 100 μl of 500 μg/ml solution of rat tail type-I collagen, peptides and heat-denatured BSA (Sigma-Aldrich) at 4 °C overnight, blocked with 100 μl of 1% heat-denatured BSA, and then washed with PBS. 100 μl of anti-integrin antibody treated and untreated HepG2 cell suspension in serum-free DMEM (10 × 10^5^ cells/ml) was added to the coated wells and incubated for 1 h at 20 °C. Nonadhered cells were removed by washing with 1× PBS (pH 7.4) two to three times. Adhered cells were counted using light microscope (33, 55).

### Immunofluorescence staining for cell spreading study

Chamber slides were coated with either rat tail type-I collagen or temporin L derived peptides and incubated overnight at 4 °C. The chamber slides were washed using 1× PBS solution three to four times. Then HepG2 cells were seeded at a density of 6 × 10^4^ cells/well in serum-free medium and allowed to adhere for 3 h at 37 °C in a humidified CO_2_ incubator. Attached cells were fixed in cold 3.7% formaldehyde for 5 to 10 min, permeabilized with 0.1% Triton X-100 for 5 min, and blocked in blocking buffer (1% BSA in MQ water) for 30 min. The actin cytoskeleton of permeabilized cells were stained with actin stress fiber specific green fluorescent dye, Alexa fluor 488-phalloidin (Invitrogen) and cell nucleus were stained by incubating the cells with Hoechst 33,342 (1 μg hoechst in 500 μl PBS/well of chamber slides) for 5 min. The slides were visualized under a Carl Zeiss instrument confocal laser scanning microscope and images were acquired at 60× magnification (33, 39, 55).

## Data availability

All the data required for this manuscript are available either in the main article or in the supporting information file.

## Supporting information

This article contains supporting information.

## Conflict of interest

The authors declare that they have no conflicts of interest with the contents of this article.
