# Supplementary material for: Aromatic residue-rich amino-terminal segments of temporin L self-assemble into collagen-mimetic peptides with cell-adhesion properties
Source: J Biol Chem. 2026 Mar 10;302(5):111356. doi: 10.1016/j.jbc.2026.111356 (PMC13125179; doi:10.1016/j.jbc.2026.111356)
Supplement: Supporting Information [file mmc1.doc]

Supporting Information

**Aromatic residue-Rich amino-terminal segments of temporin L self-Assemble into collagen-mimetic peptides with cell-adhesion Properties**

Neeraj Kumar Verma^1,2#^, Arvind Gupta^1,6#^, Malika Arora^3^, Nabanita Mukherjee^4^, Tayyaba Afshan^1^, Rahul Dev Verma^1,6^, Rahul Verma^5,6^, Jyotshana Saroj^1,6^, Garima Pant^7^, Sariyah Akhtar^1^, Surajit Ghosh^4^, Kalyan Mitra^7^, Deepa Ghosh^3^, Jimut Kanti Ghosh^1,6*^

**Running Title**: **Identification of collagen-mimetic peptides from temporin L**

^#^Authors contributed equally.

^1^Biochemistry and Structural Biology Division, CSIR-Central Drug Research Institute, Sector 10, Jankipuram Extension, Sitapur Road, Lucknow–226031, India.

^2^School of Studies In Biotechnology, Shaheed Mahendra Karma Vishwavidyalaya, Bastar, Jagdalpur-494001, Chhattisgarh, India

^3^Chemical Biology Unit, Institute of Nano Science and Technology, Knowledge City, Sector-81, Mohali-140306, Punjab, India.

^4^Smart Healthcare, Interdisciplinary Research Platform, Indian Institute of Technology, Jodhpur 342030, Rajasthan, India.

^5^CSIR-Indian Institute of Toxicology Research, Vishvigyan Bhawan, 31, Mahatma Gandhi Marg, Lucknow-226001, Uttar Pradesh, India.

^6^Academy of Scientific and Innovative Research (AcSIR), Ghaziabad, 201002, India.

^7^Electron Microscopy Unit, CSIR-Central Drug Research Institute, Lucknow, Uttar Pradesh 226031, India.

*To whom correspondence should be addressed: Jimut Kanti Ghosh, Biochemistry and Structural Biology Division, CSIR-Central Drug Research Institute, Sector 10, Jankipuram Extension, Sitapur Road, Lucknow–226 031, India, E-mail: jighosh@yahoo.com

1. **Supplementary Figure**


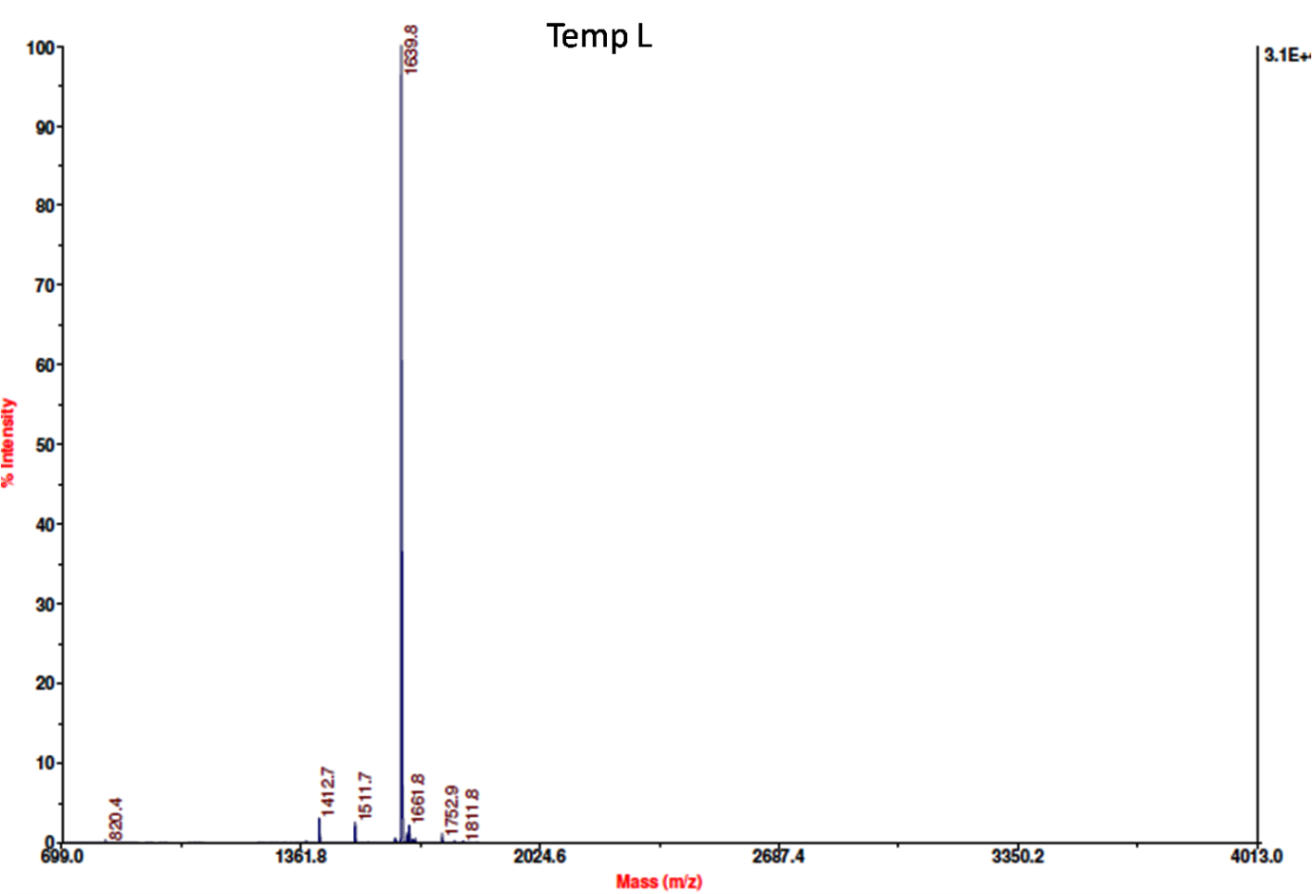


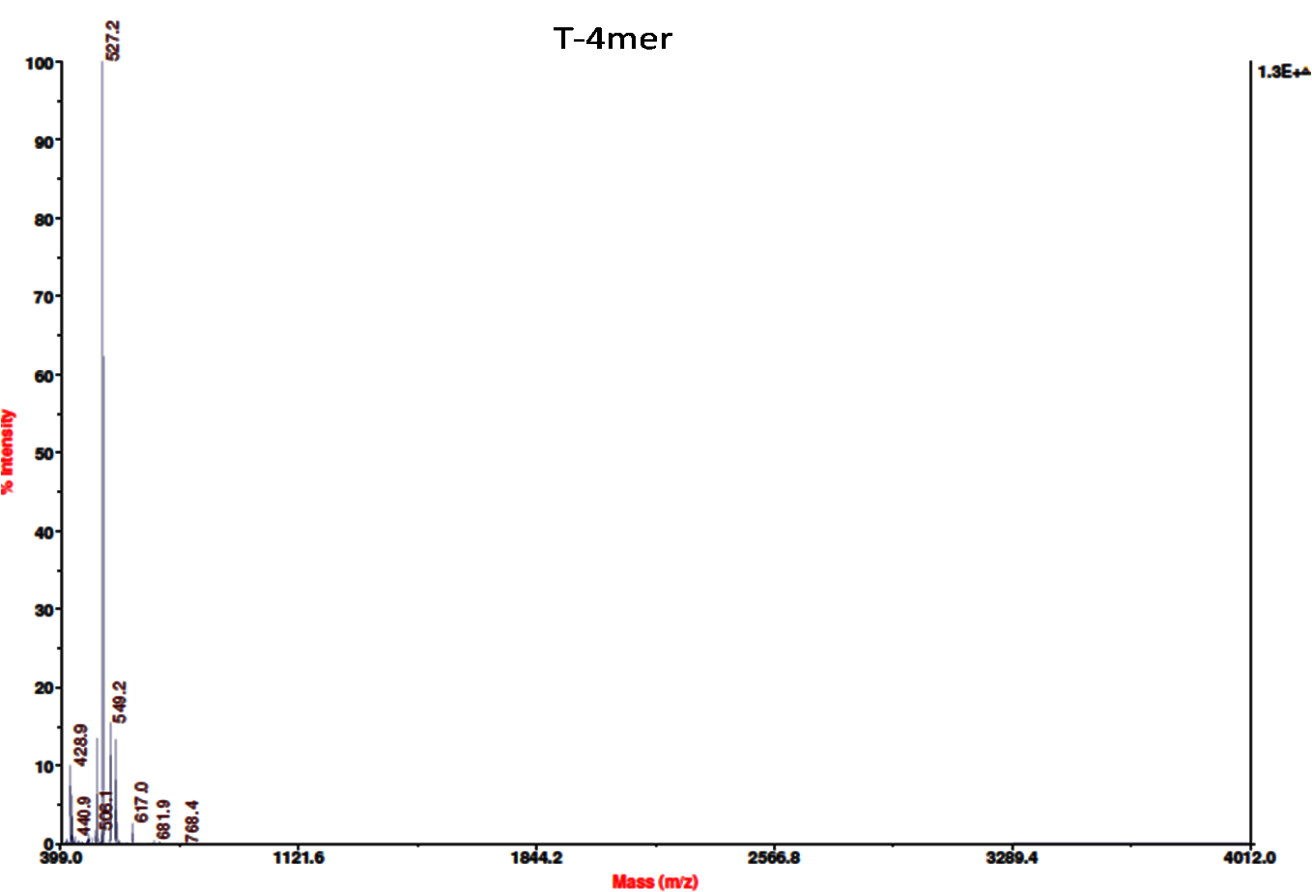


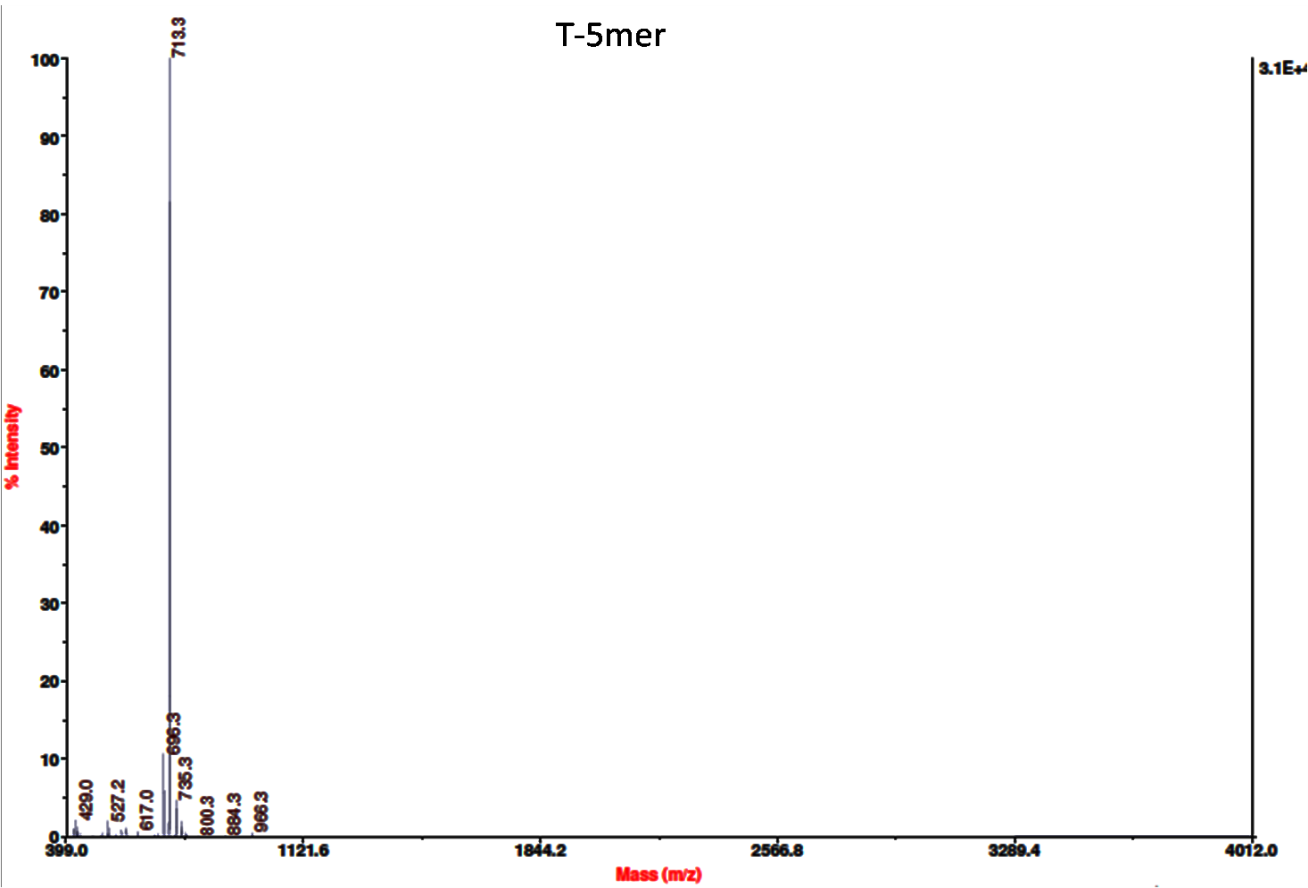


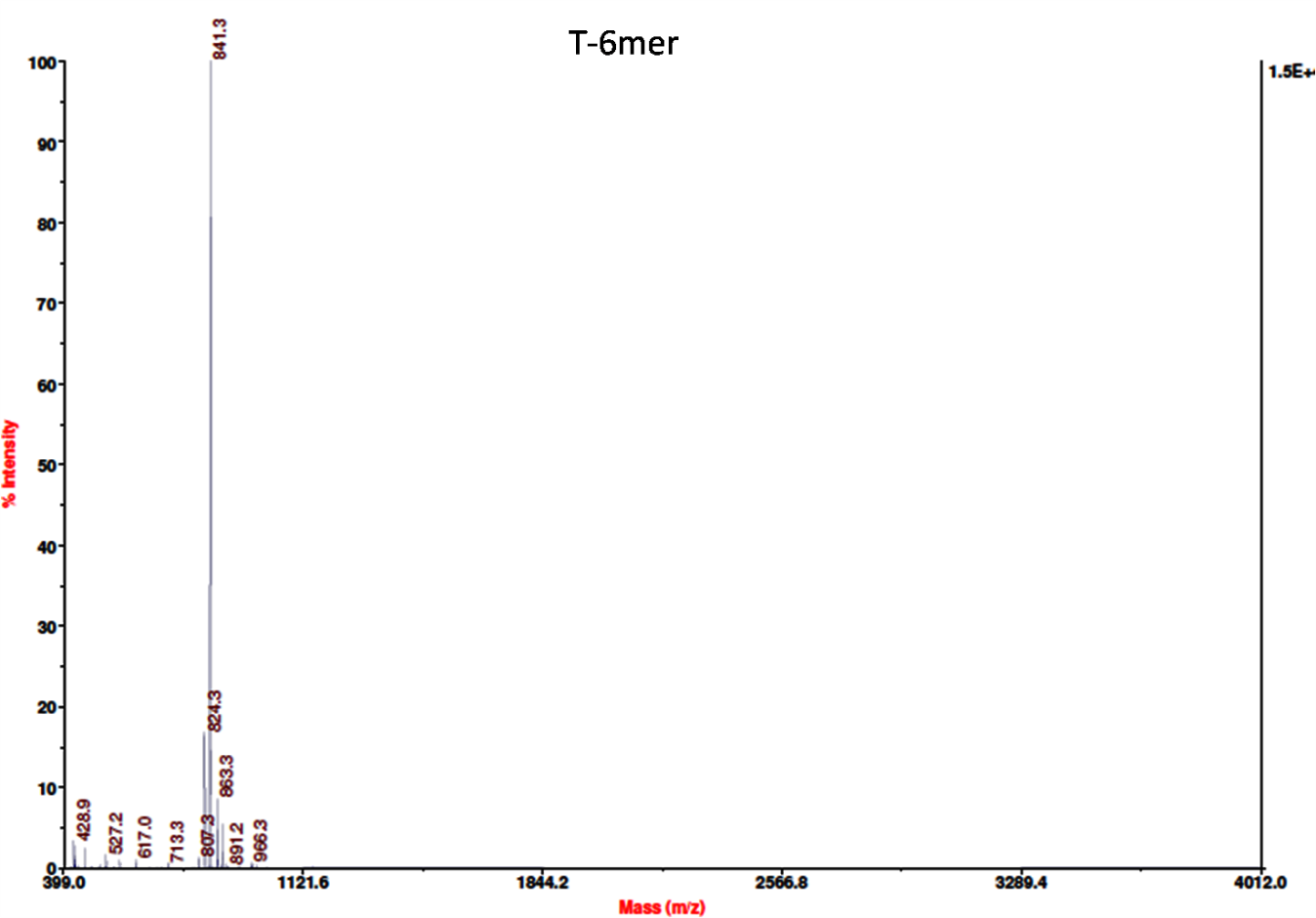


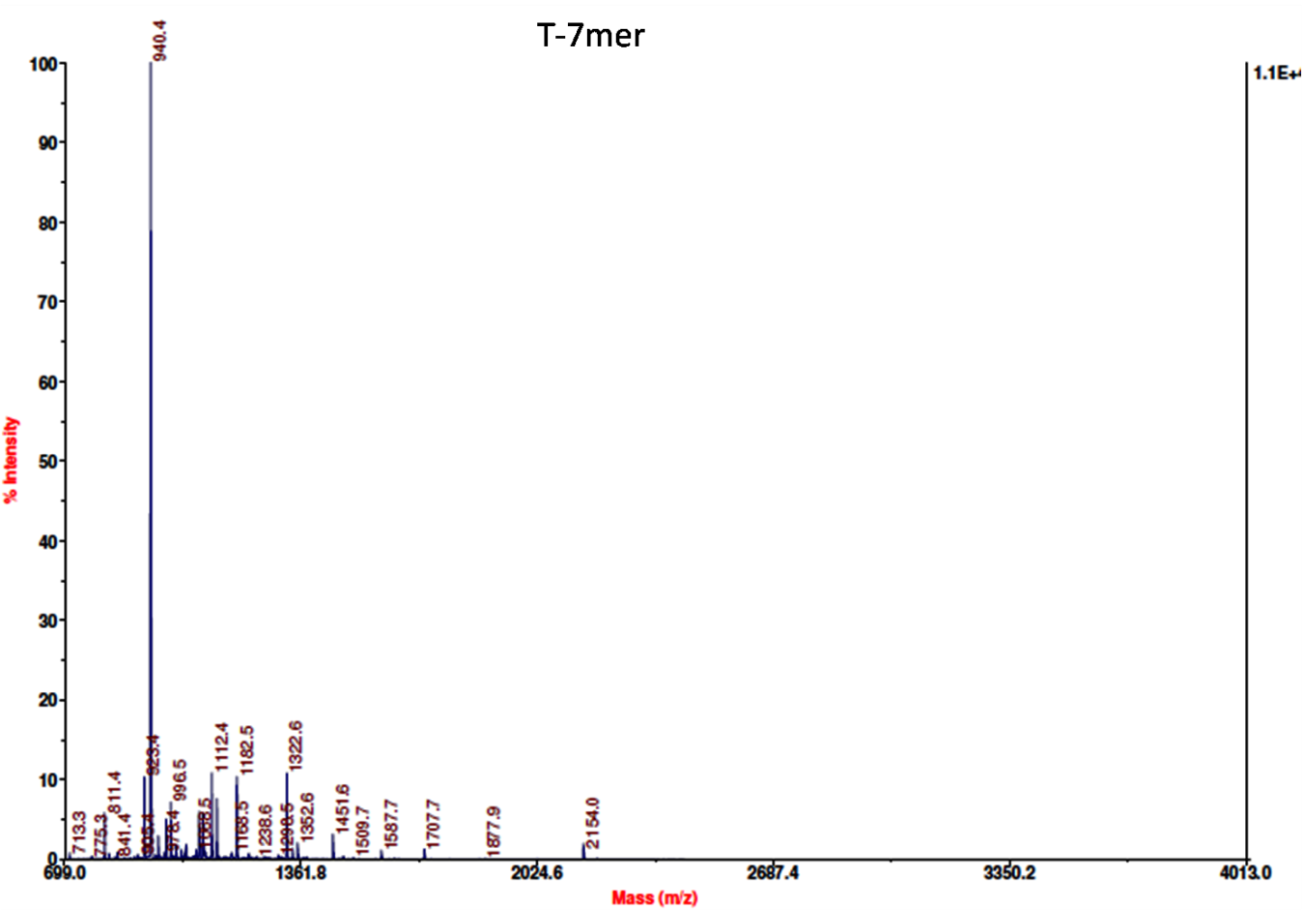


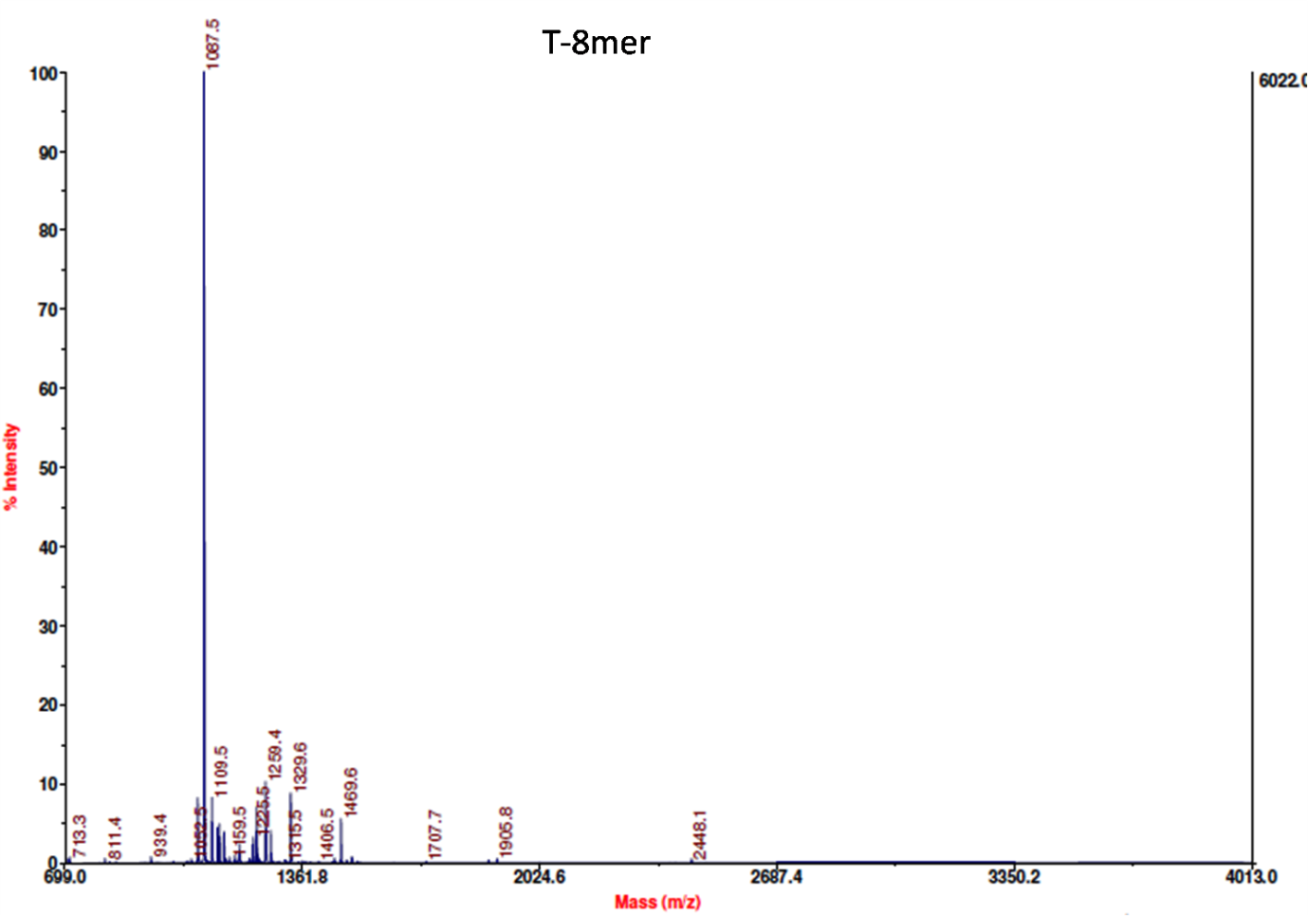


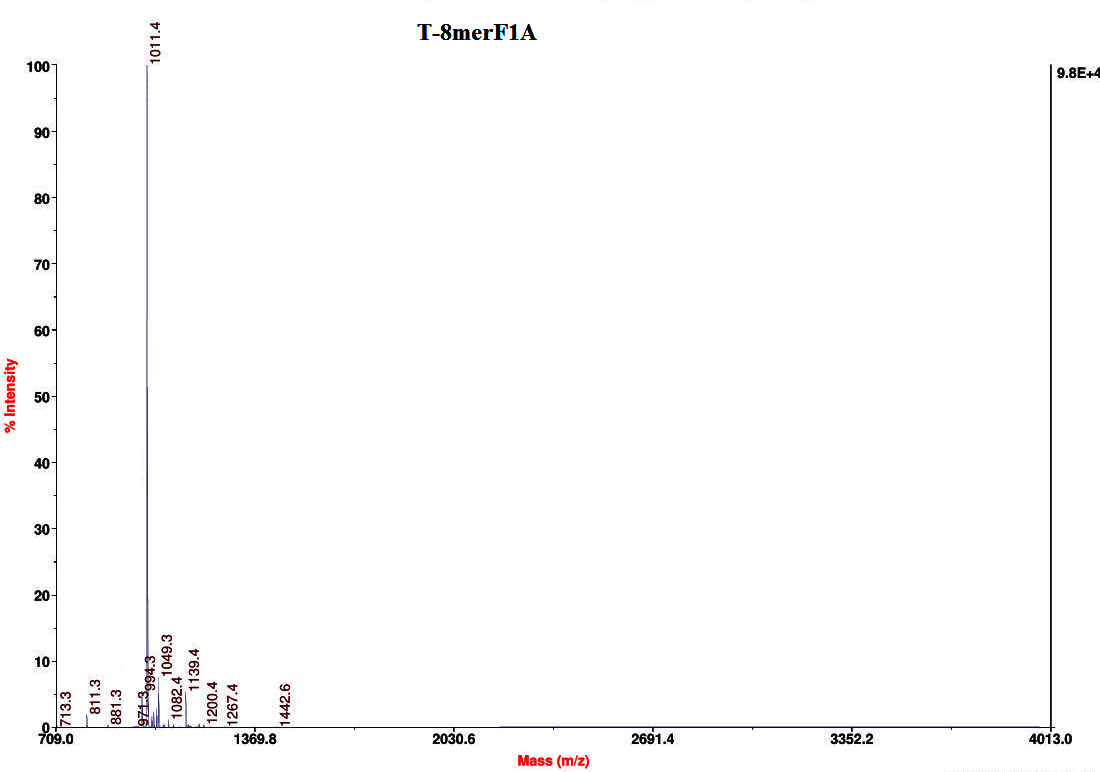


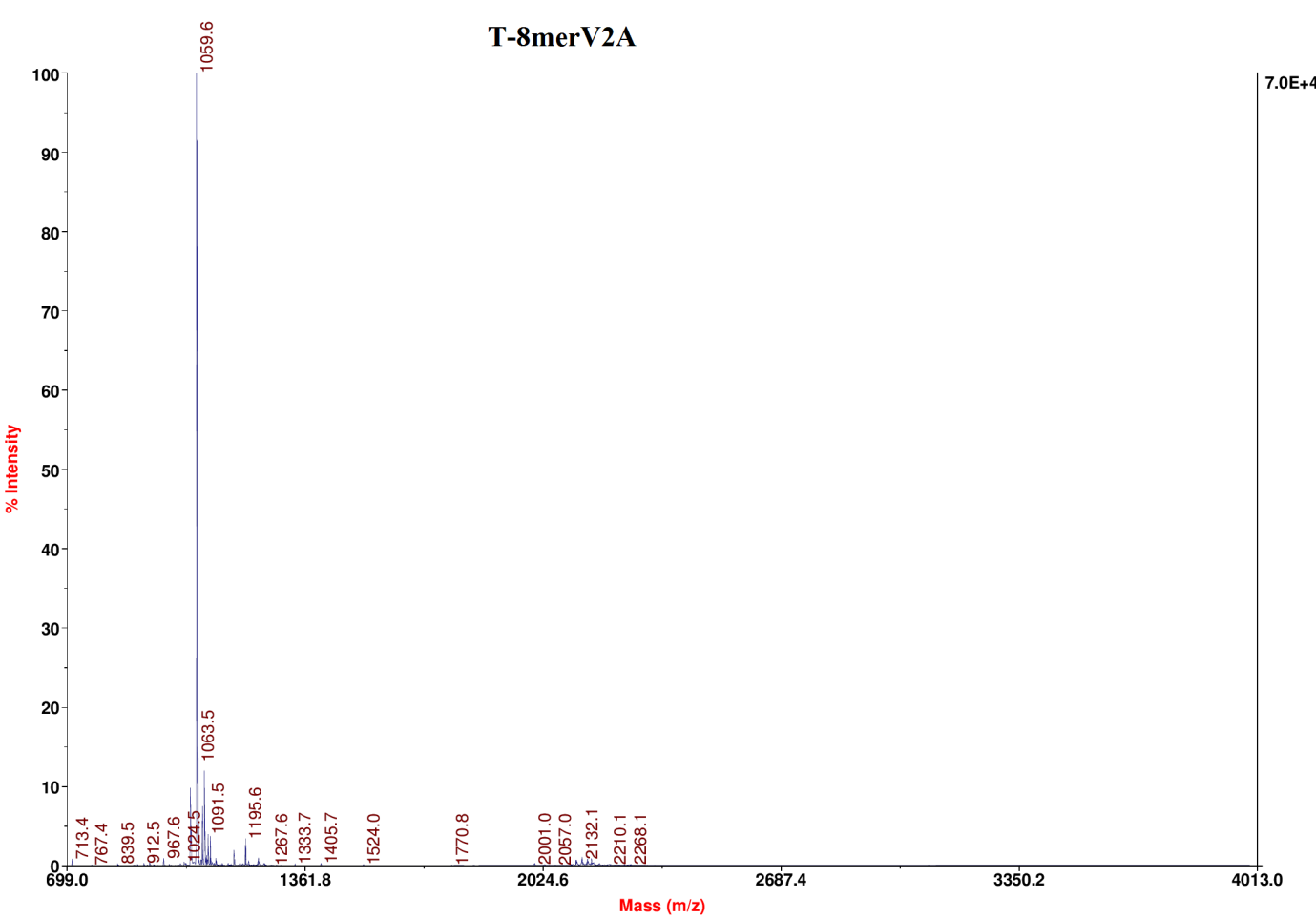


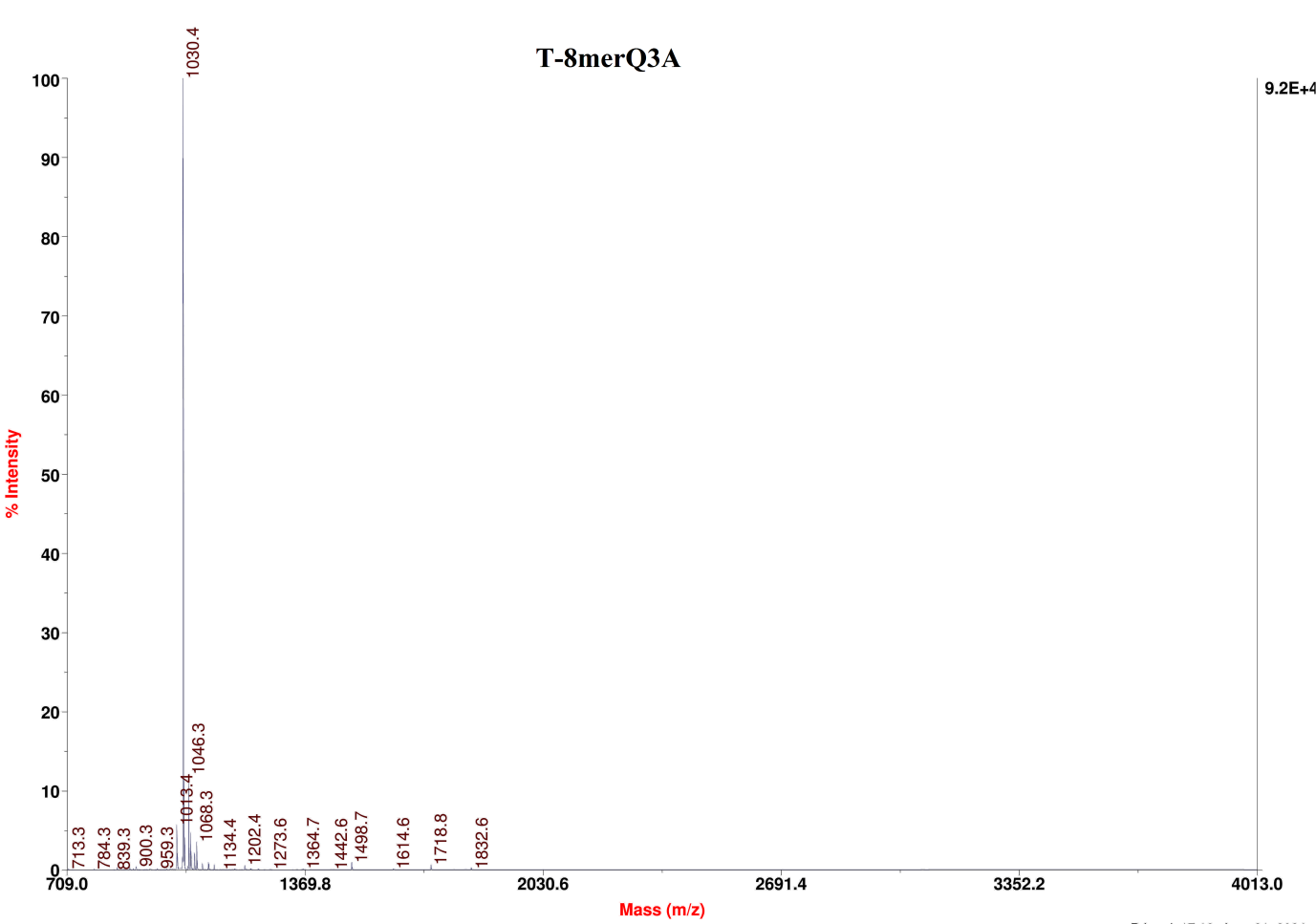


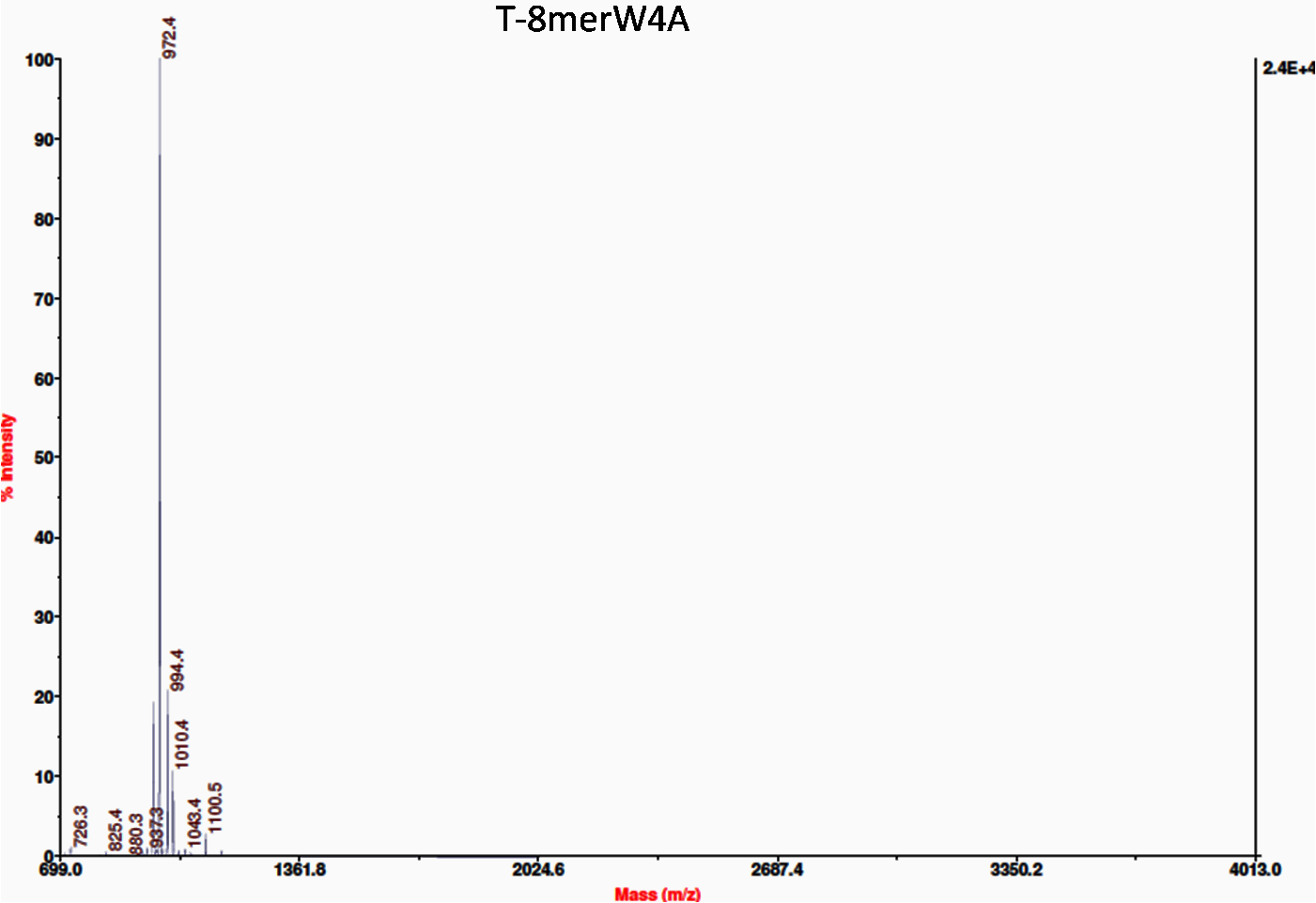


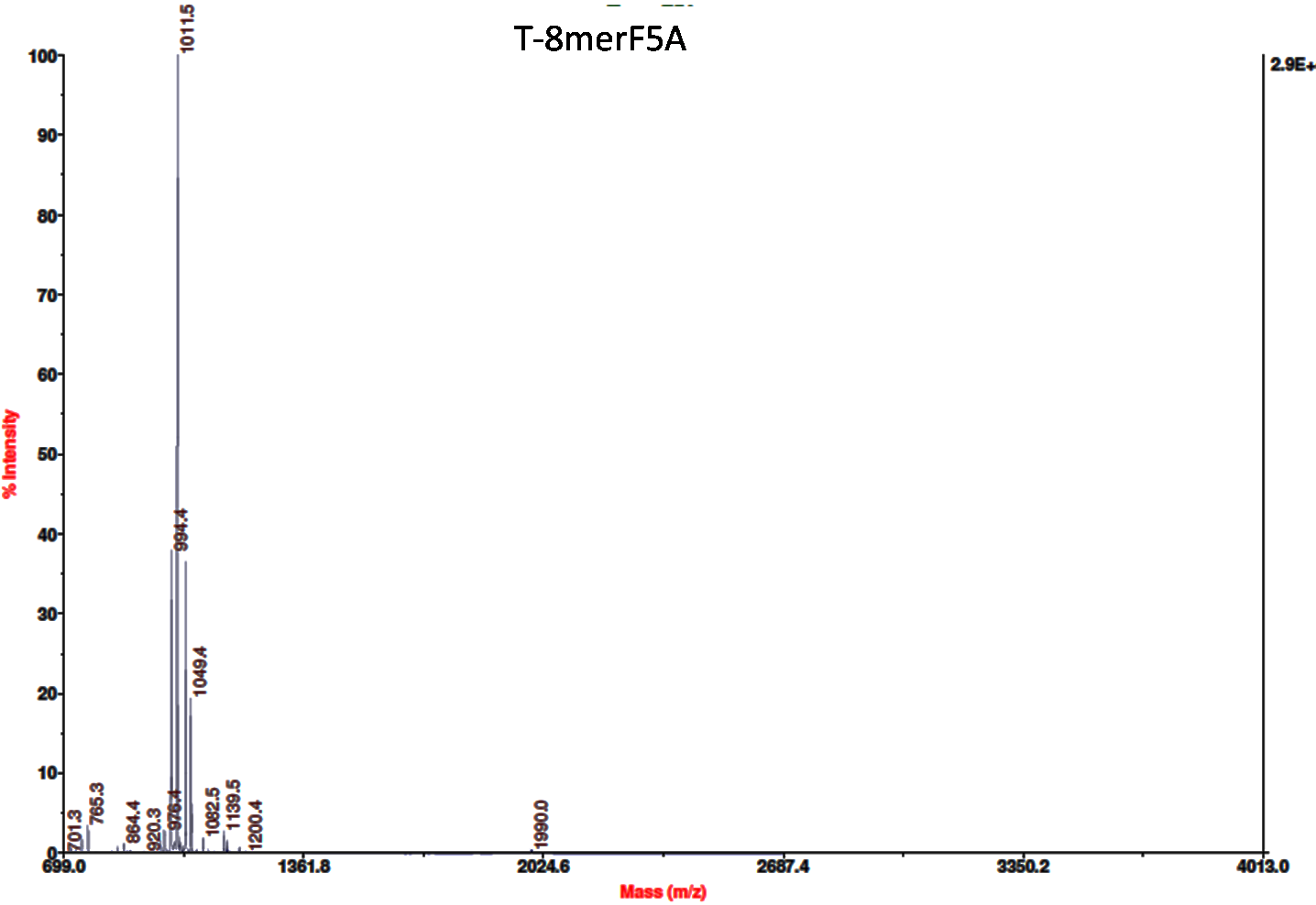


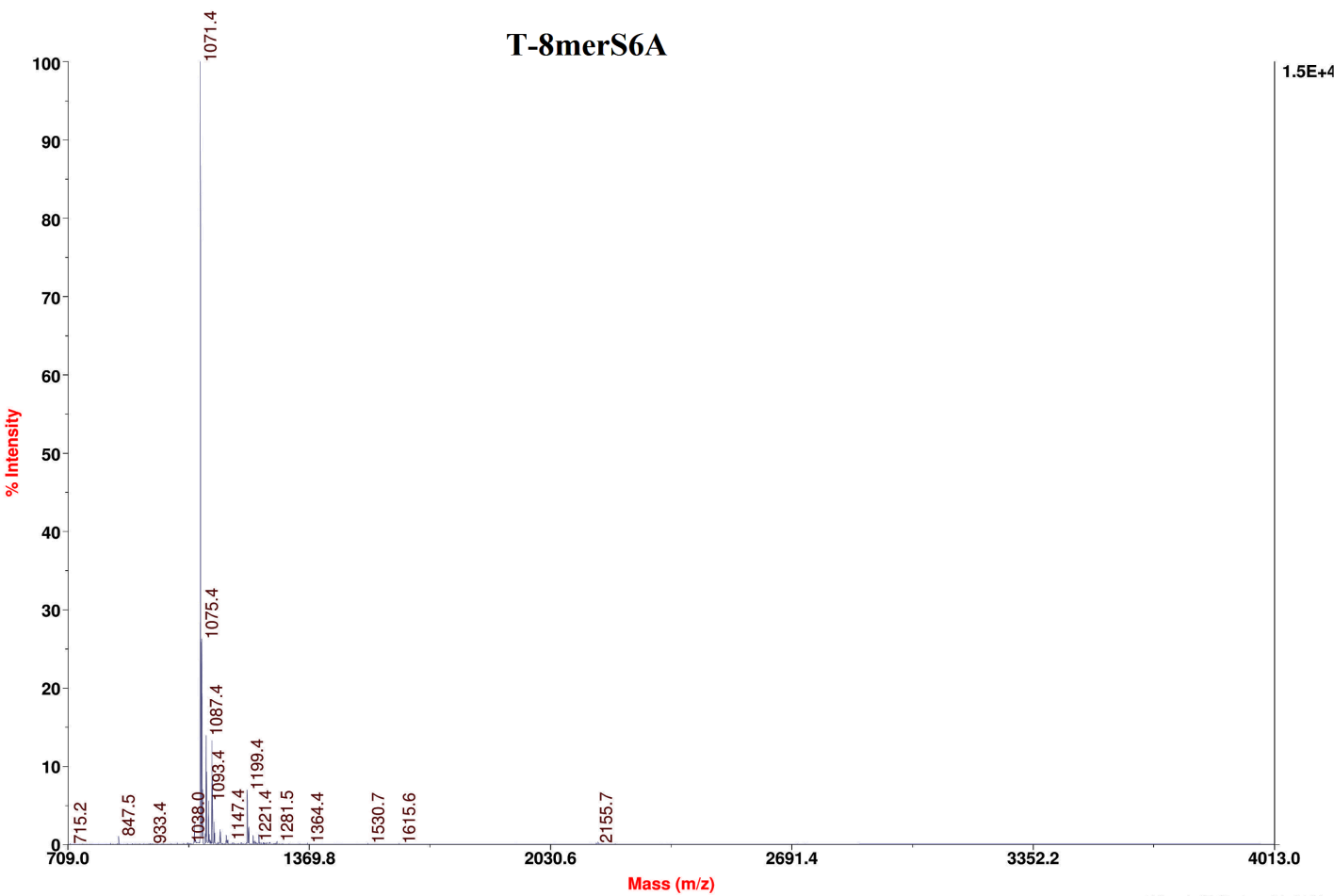


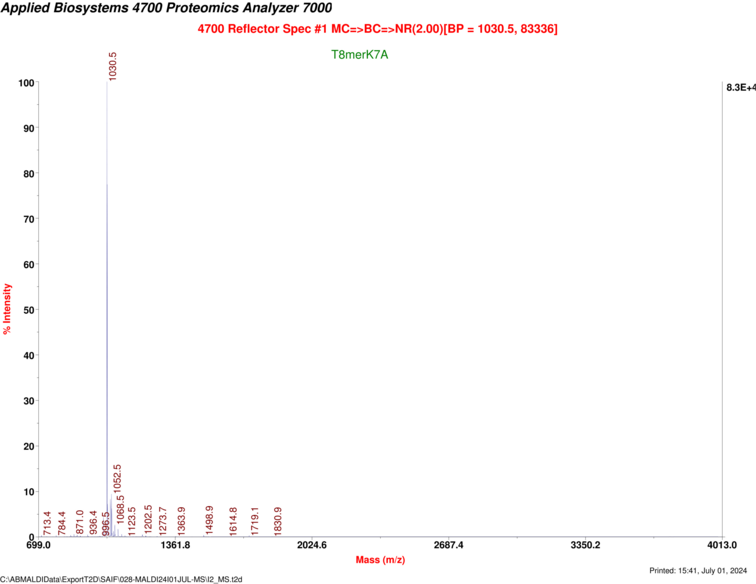


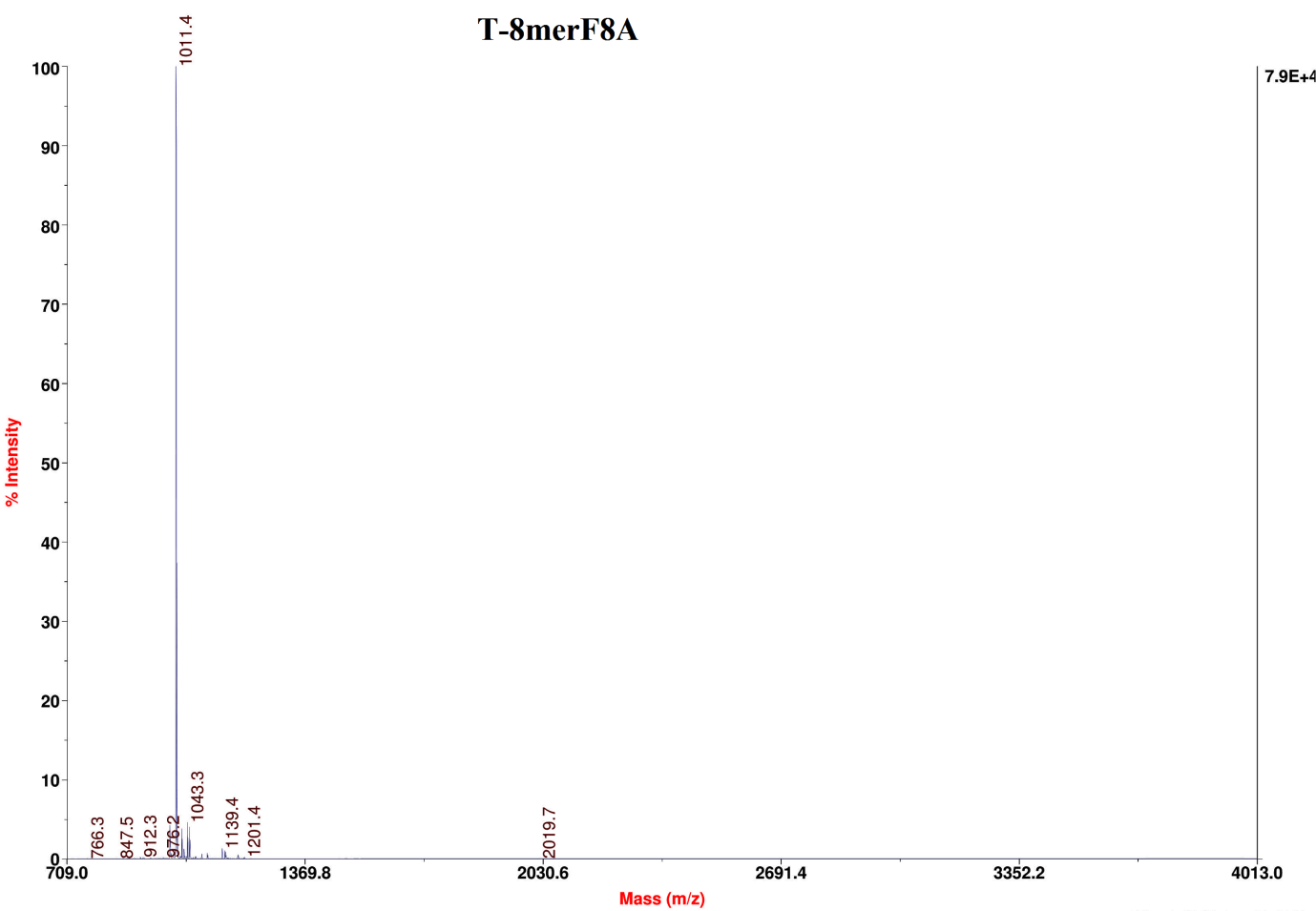


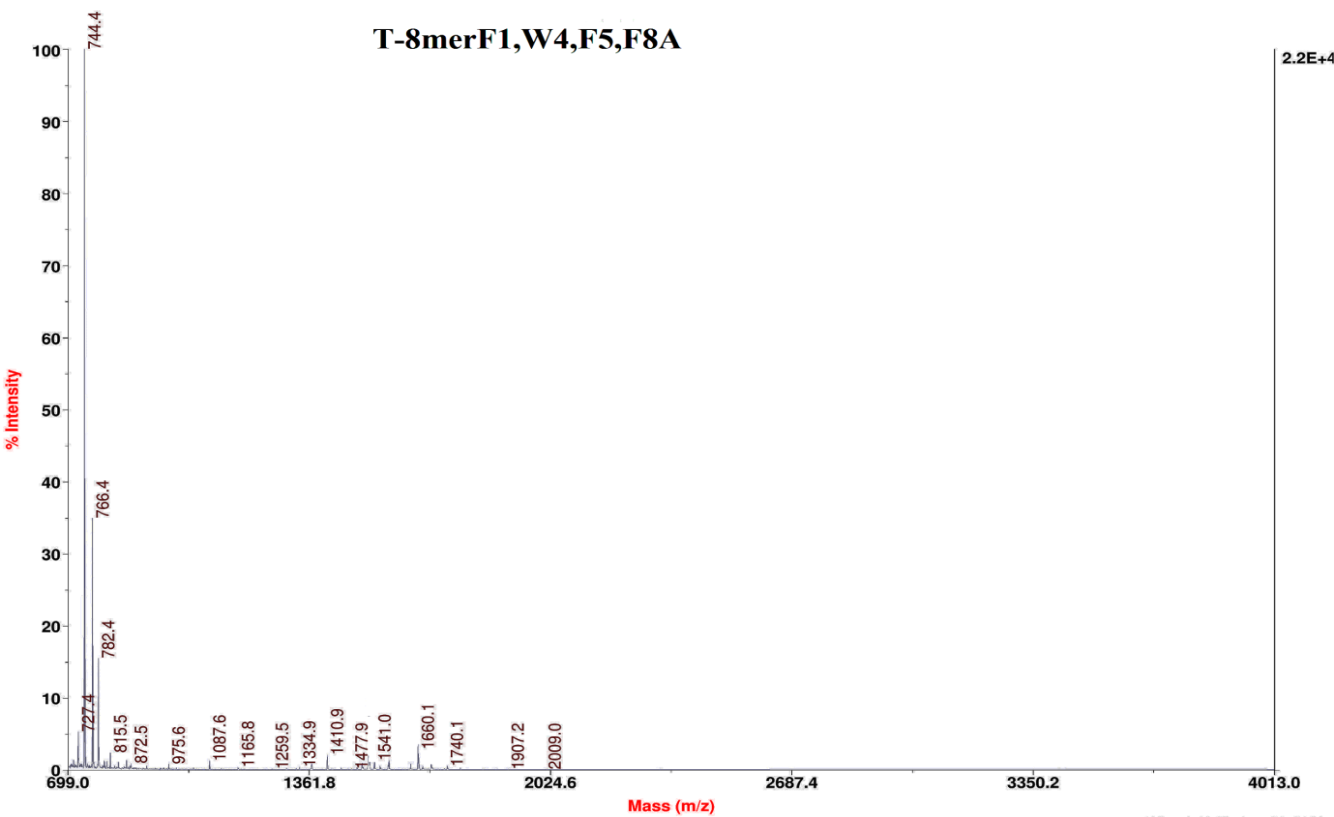


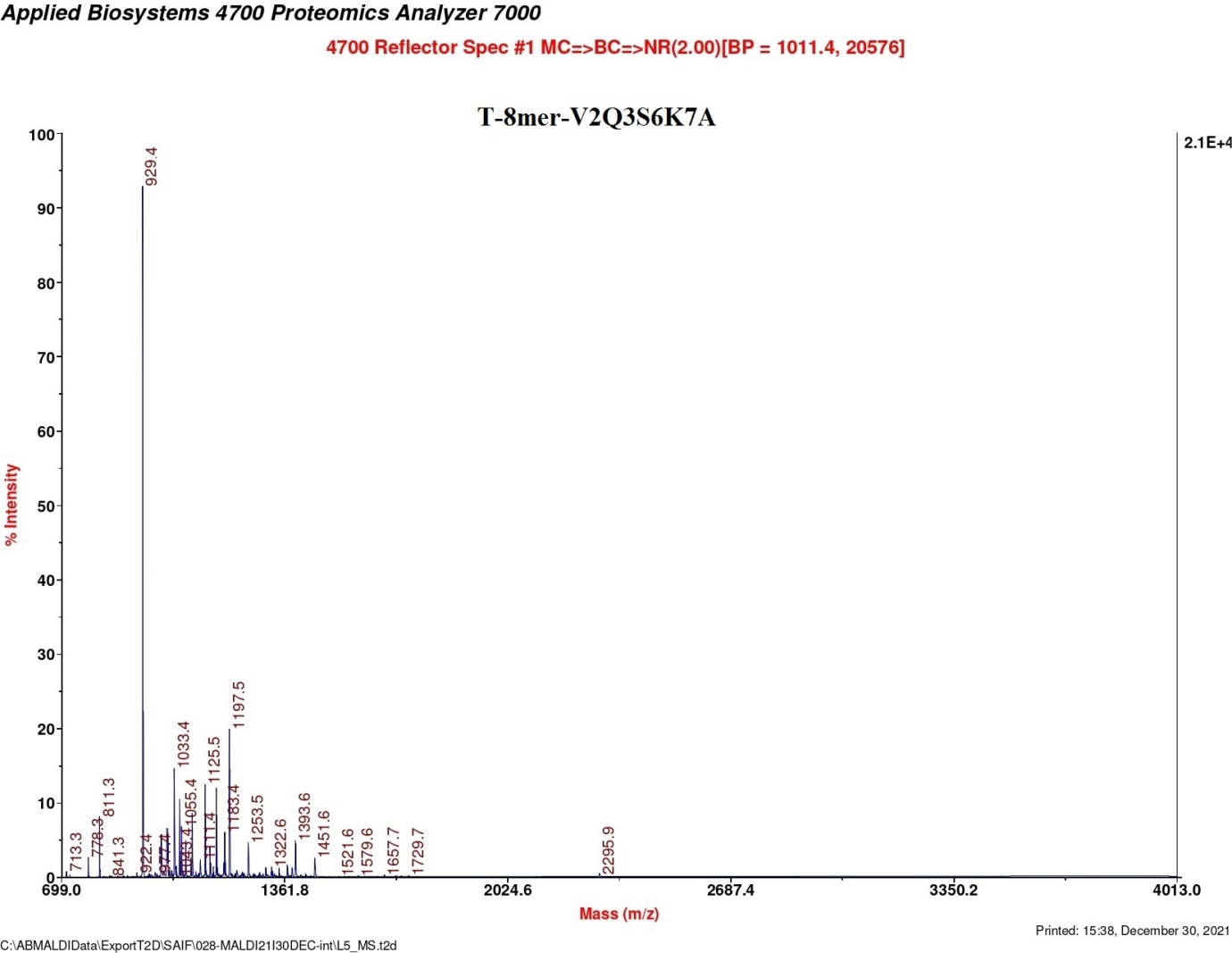


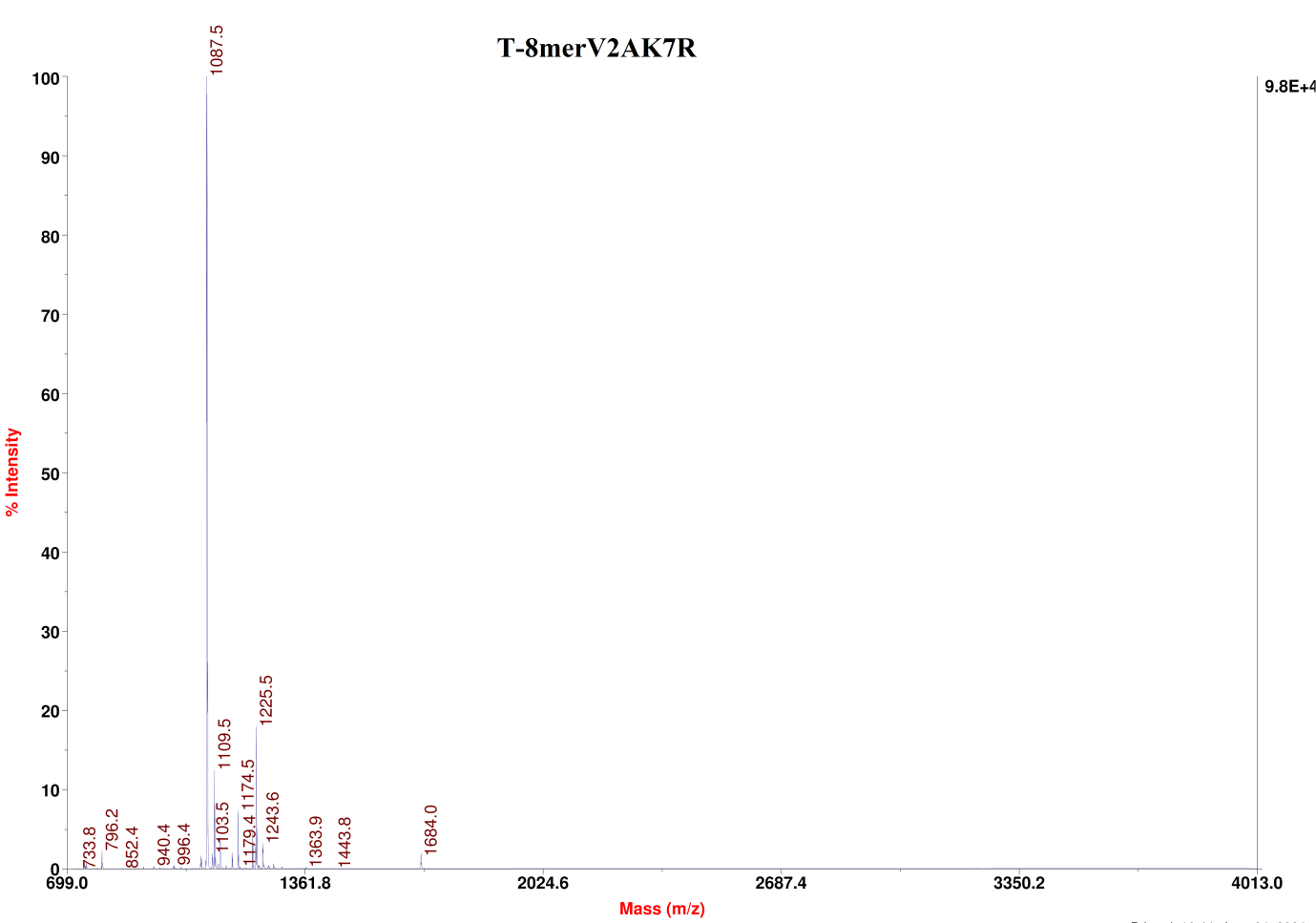


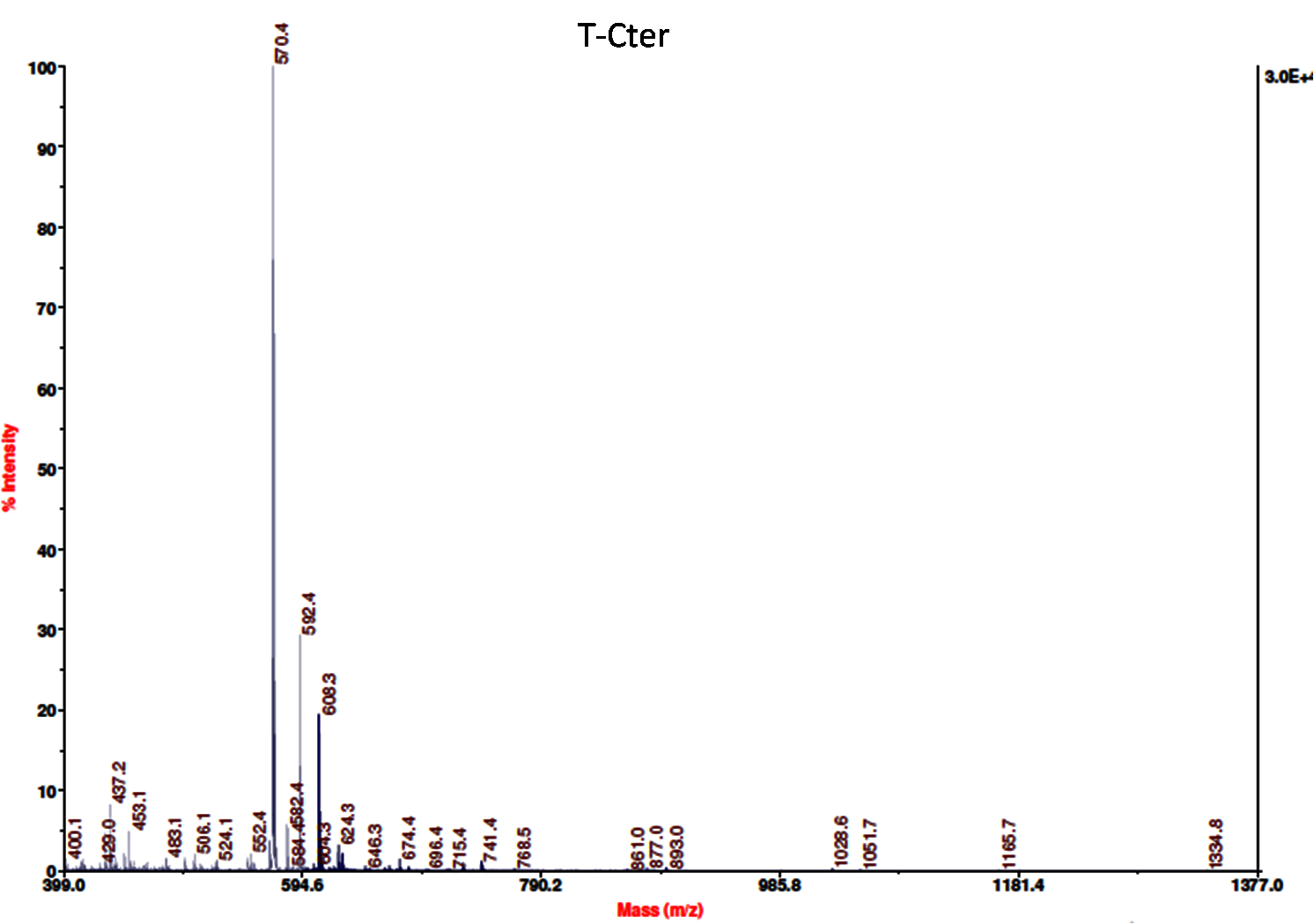


Figure S1. MALDI-TOF mass spectra of the peptides.

**Blank**

**Temp L**

| **S. No.** | **Retention Time** | **Area** | **% Area** |
| --- | --- | --- | --- |
| 1 | 19.572 | 233908 | 0.88 |
| 2 | 21.496 | 825814 | 3.12 |
| 3 | 23.275 | 25428570 | 96.00 |

**T-4mer**

| **S. No.** | **Retention Time** | **Area** | **% Area** |
| --- | --- | --- | --- |
| 1 | 10.501 | 15996819 | 97.17 |
| 2 | 11.497 | 280537 | 1.70 |
| 3 | 12.010 | 186173 | 1.13 |

**T-5mer**

| **S. No.** | **Retention Time** | **Area** | **% Area** |
| --- | --- | --- | --- |
| 1 | 14.730 | 8765231 | 99.60 |
| 2 | 17.657 | 35072 | 0.40 |

**T-6mer**

| **S. No.** | **Retention Time** | **Area** | **% Area** |
| --- | --- | --- | --- |
| 1 | 15.492 | 45613467 | 99.20 |
| 2 | 16.450 | 368224 | 0.80 |

**T-7mer**

| **S. No.** | **Retention Time** | **Area** | **% Area** |
| --- | --- | --- | --- |
| 1 | 15.767 | 26286 | 0.13 |
| 2 | 16.193 | 19865226 | 99.87 |

**T-8mer**

| **S. No.** | **Retention Time** | **Area** | **% Area** |
| --- | --- | --- | --- |
| 1 | 17.233 | 189732 | 0.57 |
| 2 | 18.186 | 32951284 | 99.43 |

**T-8mer F1A**

| **S. No.** | **Retention Time** | **Area** | **% Area** |
| --- | --- | --- | --- |
| 1 | 17.011 | 4137251 | 99.51 |
| 2 | 17.733 | 20482 | 0.49 |

**T-8mer V2A**

| **S. No.** | **Retention Time** | **Area** | **% Area** |
| --- | --- | --- | --- |
| 1 | 16.976 | 33188763 | 99.49 |
| 2 | 20.055 | 170185 | 0.51 |

**T-8mer Q3A**

| **S. No.** | **Retention Time** | **Area** | **% Area** |
| --- | --- | --- | --- |
| 1 | 18.707 | 11554410 | 98.44 |
| 2 | 19.559 | 183270 | 1.56 |

**T-8mer W4A**

| **S. No.** | **Retention Time** | **Area** | **% Area** |
| --- | --- | --- | --- |
| 1 | 14.421 | 28305398 | 99.20 |
| 2 | 15.050 | 228623 | 0.80 |

**T-8mer F5A**

| **S. No.** | **Retention Time** | **Area** | **% Area** |
| --- | --- | --- | --- |
| 1 | 15.093 | 98522 | 0.87 |
| 2 | 15.352 | 11278426 | 99.13 |

**T-8mer S6A**

| **S. No.** | **Retention Time** | **Area** | **% Area** |
| --- | --- | --- | --- |
| 1 | 12.298 | 204512 | 3.37 |
| 2 | 18.484 | 5787263 | 95.26 |
| 3 | 19.326 | 83358 | 1.37 |

**T-8mer K7A**

| **S. No.** | **Retention Time** | **Area** | **% Area** |
| --- | --- | --- | --- |
| 1 | 20.201 | 7071299 | 99.23 |
| 2 | 20.867 | 54685 | 0.77 |

**T-8mer F8A**

| **S. No.** | **Retention Time** | **Area** | **% Area** |
| --- | --- | --- | --- |
| 1 | 15.709 | 12286535 | 100.00 |

**T-8mer-F1,W4,F5,F8A**

| **S. No.** | **Retention Time** | **Area** | **% Area** |
| --- | --- | --- | --- |
| 1 | 6.010 | 12249003 | 100.00 |


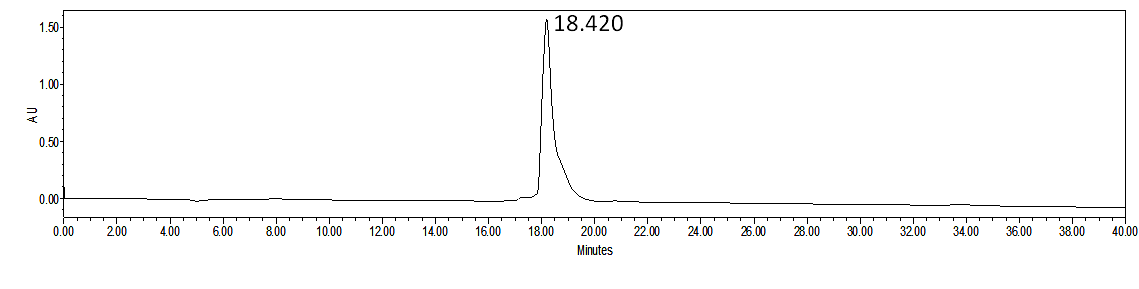


**T-8mer V2Q3S6K7A**

| **S. No.** | **Retention Time** | **Area** | **% Area** |
| --- | --- | --- | --- |
| 1 | 18.420 | 22842282 | 100.00 |

**T-8mer V2A, K7R**

**T-Cter-5mer**

| **S. No.** | **Retention Time** | **% Area** |
| --- | --- | --- |
| 1 | 12.640 | 100.00 |

**Figure S2.** HPLC purity profiles of the peptides.

**
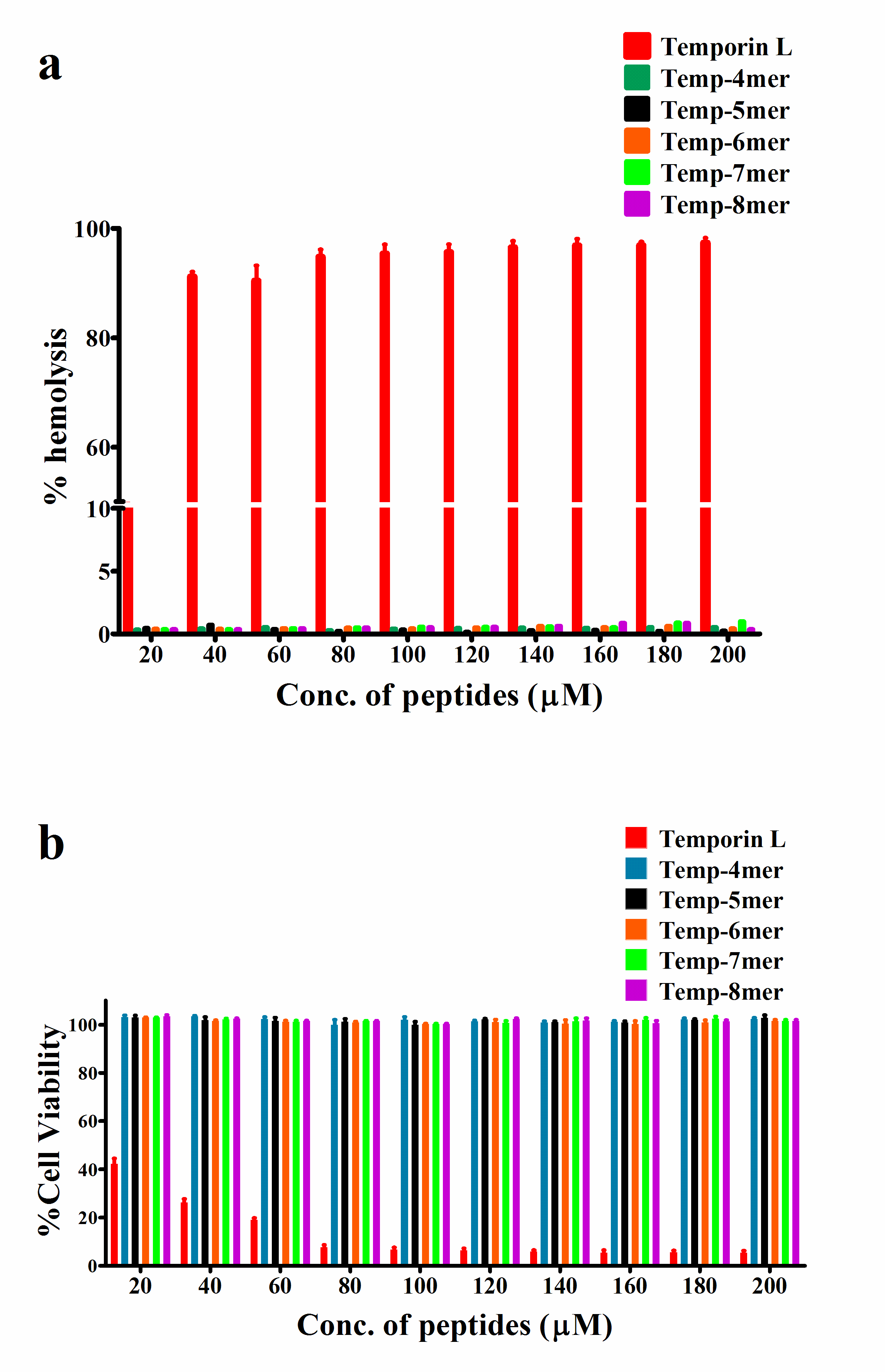
**

**Figure S3. Haemolytic activity and cytotoxic property studies of the peptides. a,** haemolytic activity assay of the peptides against hRBCs. **b**, Determination of cytotoxicity of the peptides by MTT assay showing the percent cell viability of peptide-treated HepG2 cells.


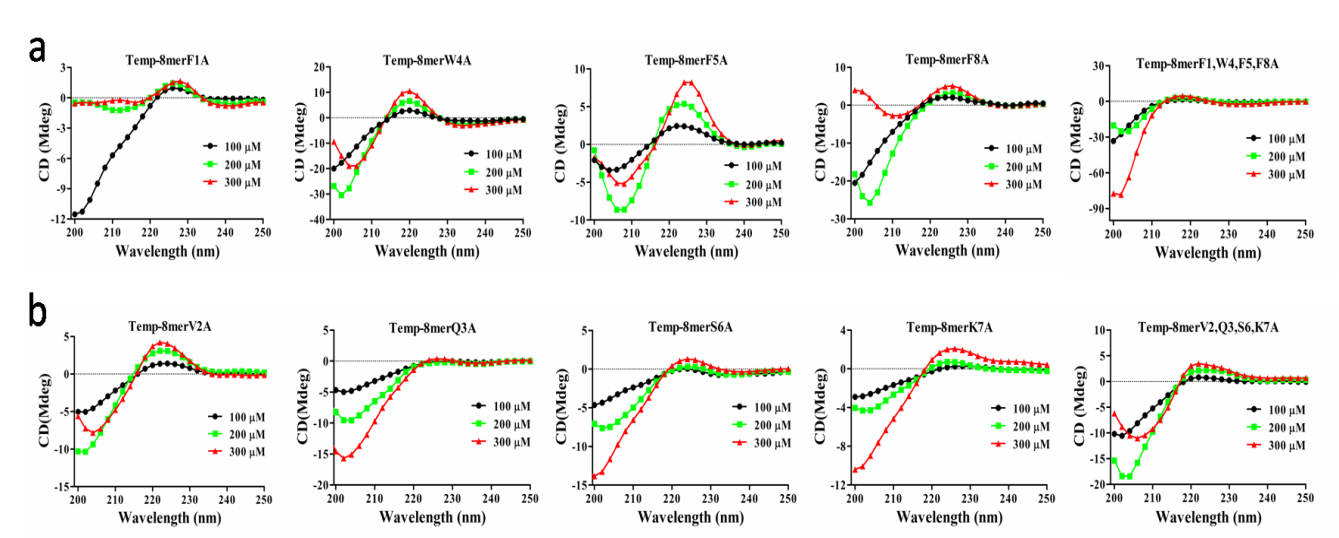


**
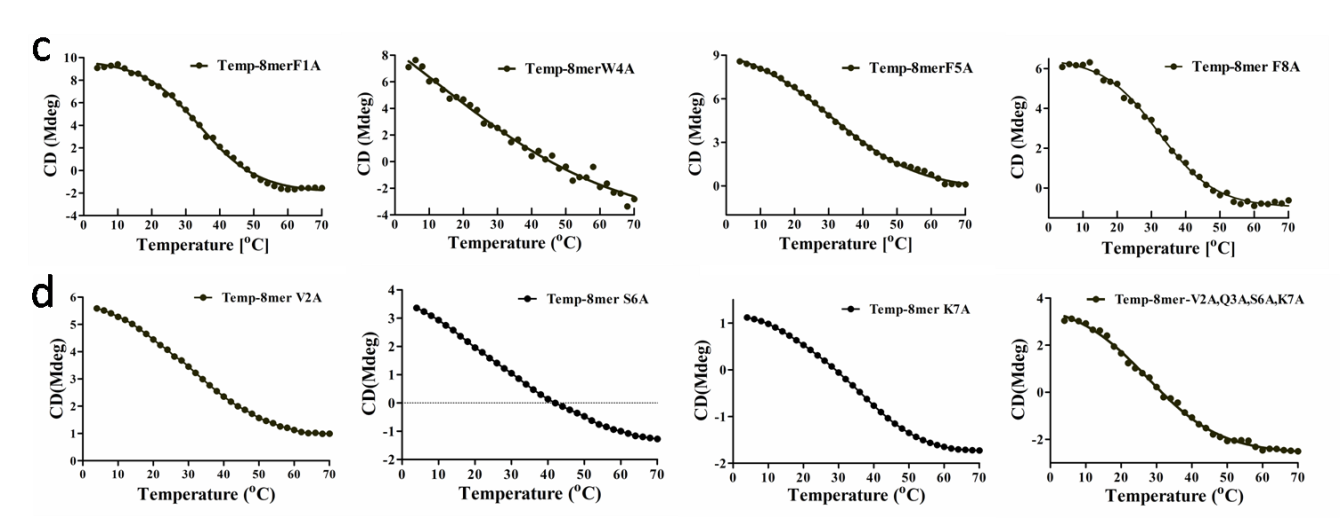
**

**Figure S4. Circular dichroism study of alanine-substituted T-8mer analogs.** CD spectra of T-8mer analogs **a**, with substitution of aromatic amino acid residues with alanine residues and **b,** with substitution of aliphatic amino acid residues with alanine residues. Panel **c** and **d** showing the melting curves of alanine substituted T-8mer analogs. The concentration of peptides was 300 μM in MQ water (pH 7.4)

.

**
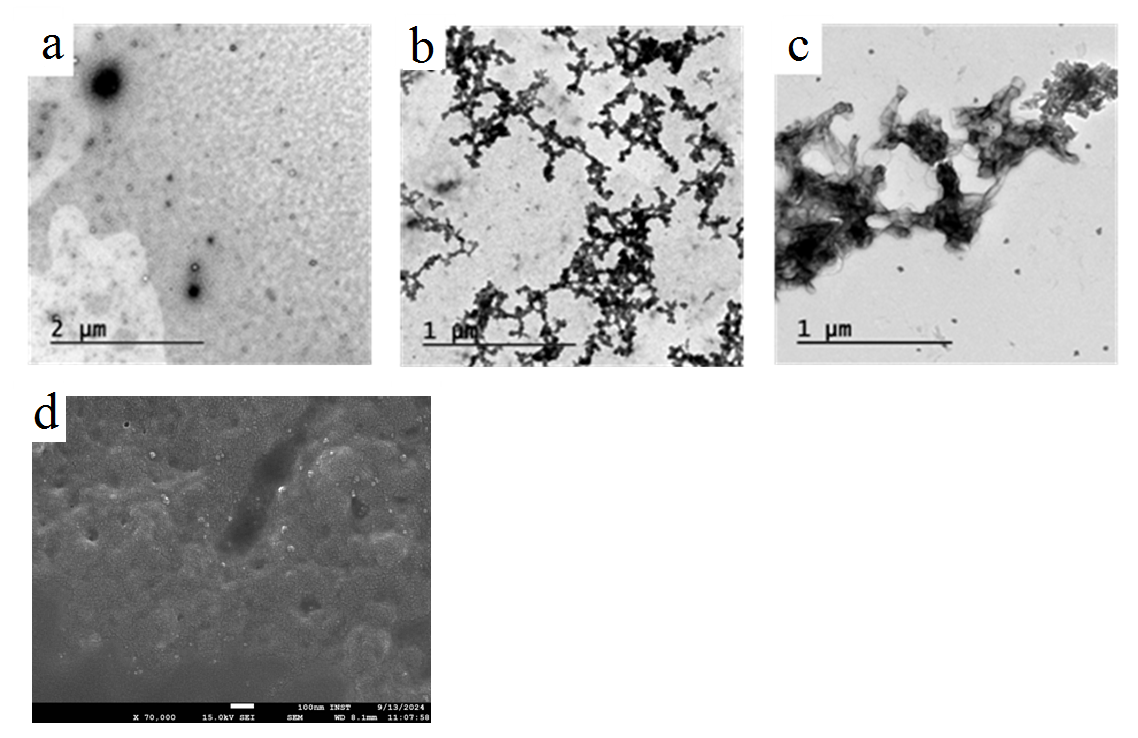
**

**Figure S5.** Representative TEM micrographs of **a)** TempL, **b)** T-4mer and **c)** T-5mer.


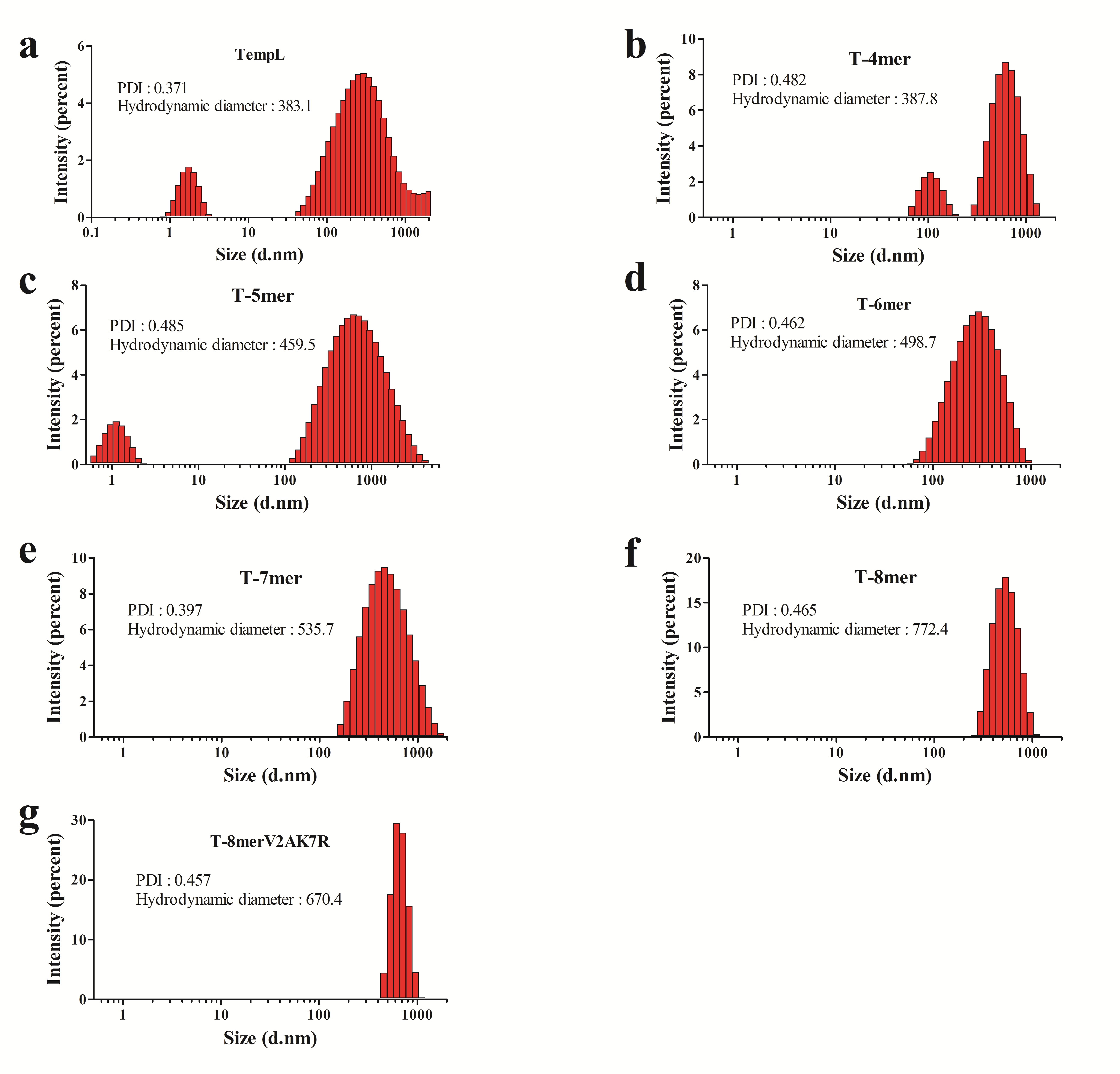


**Figure S6**. **Size distribution of nanostructures formed by** a) TempL, b) T-4mer, c) T-5mer, d) T-6mer, e) T-7mer, f) T-8mer and g) T-8merV2AK7R, as determined by Dynamic Light Scattering measurements. Respective PDI (polydispersity index) and hydrodynamic diameter for each peptide is shown. All the tested peptides were diluted to 0.02% (w/v) in MQ water and followed by standing at room temperature for 1 h before measurements.

**
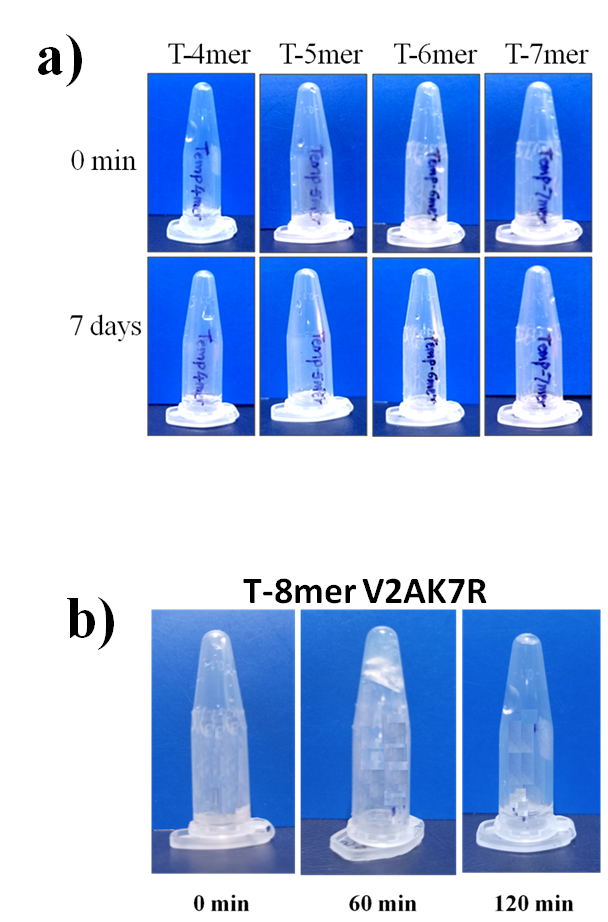
**

**Figure S7.** Inverted test tube study for examination of hydrogel-formation by the peptides.


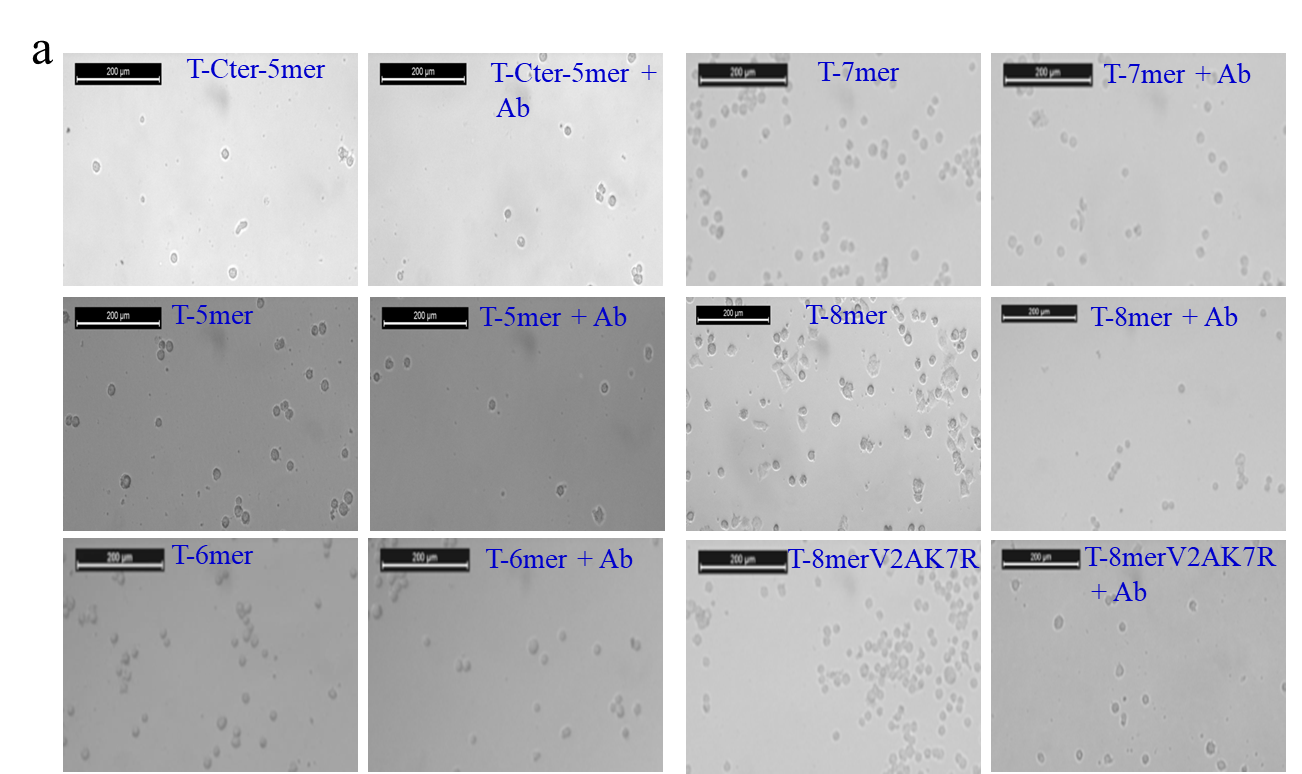


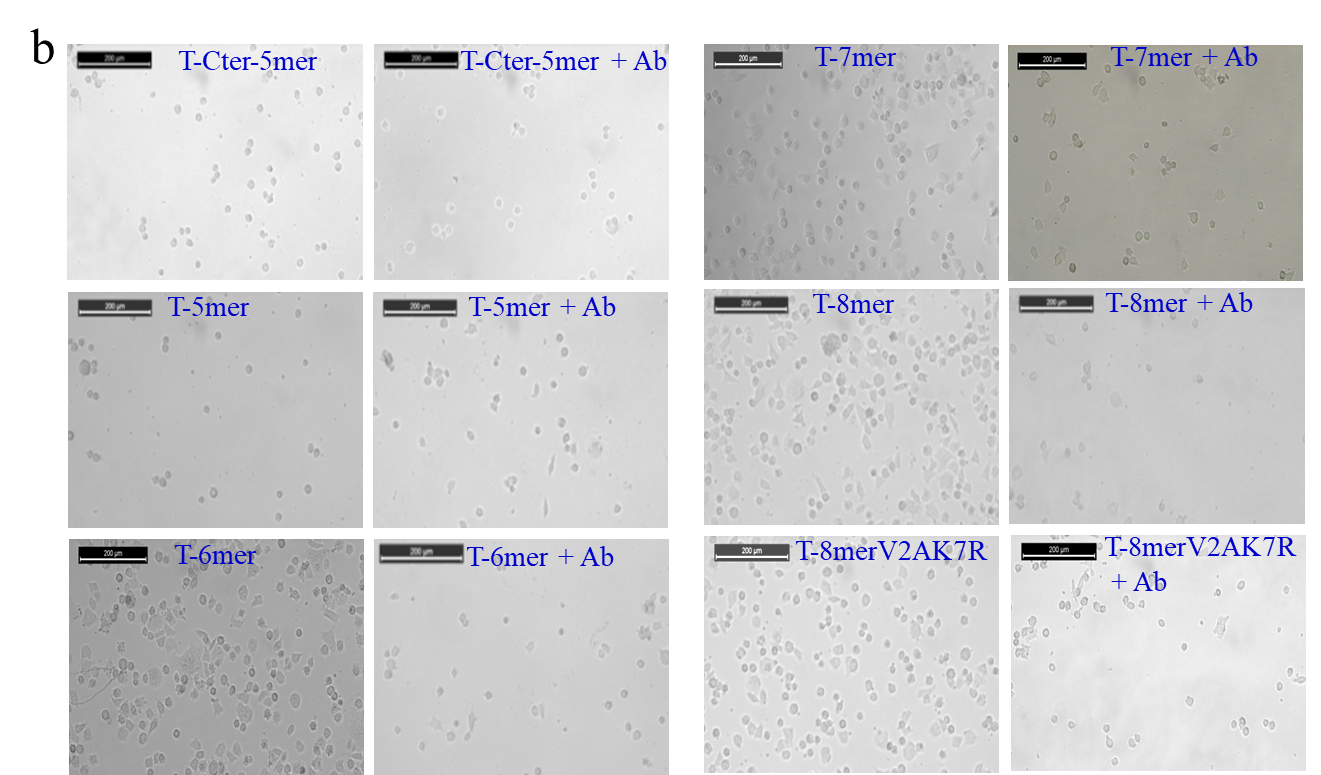


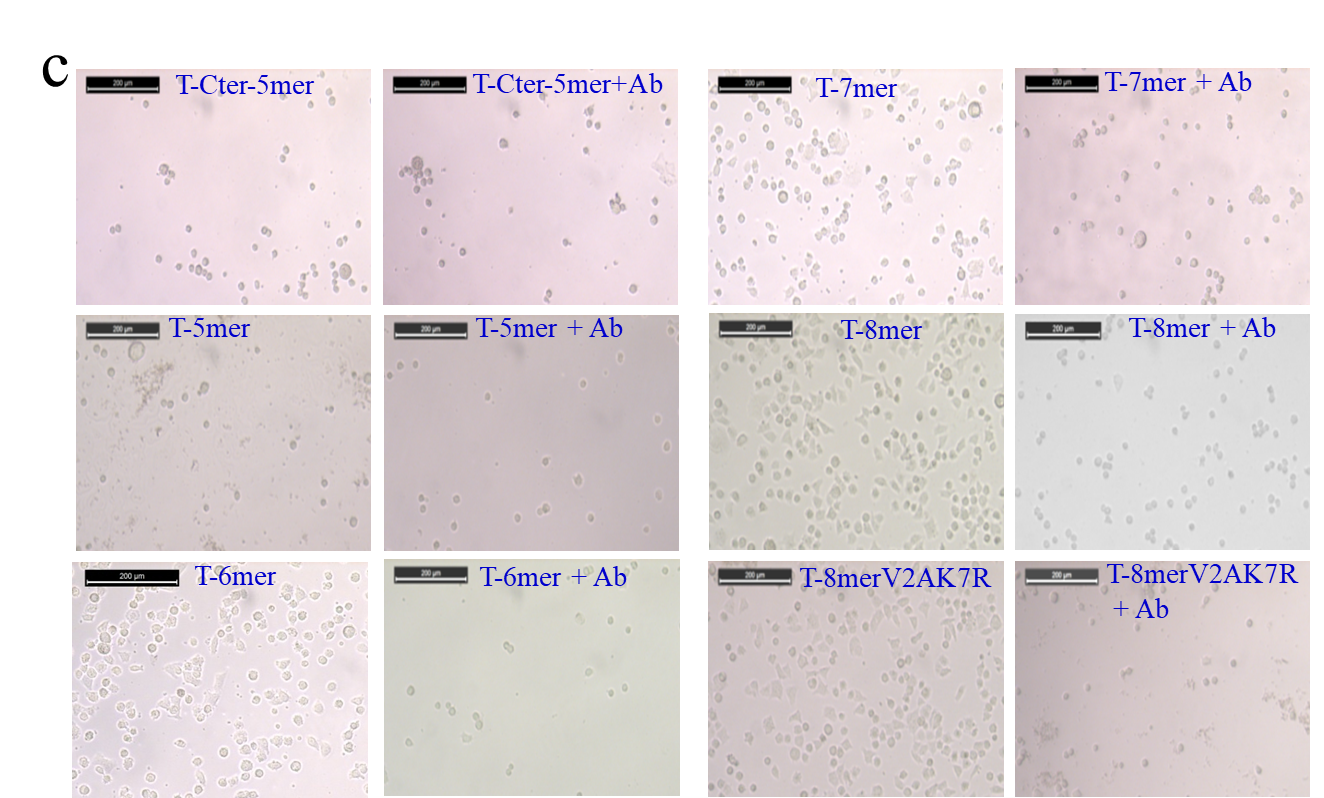


**Figure S8.** Light microscopic images showing the anti-integrin Ab untreated and anti-integrin Ab pretreated adhered cells onto **a)** 10 μg/ml **b)** 25 μg/ml and **c)** 100 μg/ml concentrations of different TempL-derived peptides coated surface.

**References:**

1. Fields, G. B.; Noble, R. L., Solid phase peptide synthesis utilizing 9-fluorenylmethoxycarbonyl amino acids. *Int J Pept Protein Res* **1990,** *35* (3), 161-214.

2. Albericio, F.; Carpino, L. A., Coupling reagents and activation. *Methods Enzymol* **1997,** *289*, 104-26.

3. Kaiser, E.; Colescott, R. L.; Bossinger, C. D.; Cook, P. I., Color test for detection of free terminal amino groups in the solid-phase synthesis of peptides. *Anal Biochem* **1970,** *34* (2), 595-8.

4. Oren, Z.; Shai, Y., Selective lysis of bacteria but not mammalian cells by diastereomers of melittin: structure-function study. *Biochemistry* **1997,** *36* (7), 1826-35.

5. Kumari, T.; Verma, D. P.; Afshan, T.; Verma, N. K.; Pant, G.; Ali, M.; Shukla, P. K.; Mitra, K.; Ghosh, J. K., A Noncytotoxic Temporin L Analogue with In Vivo Antibacterial and Antiendotoxin Activities and a Nonmembrane-Lytic Mode of Action. *ACS Infect Dis* **2020,** *6* (9), 2369-2385.

6. Lee, J. K.; Seo, C. H.; Luchian, T.; Park, Y., Antimicrobial Peptide CMA3 Derived from the CA-MA Hybrid Peptide: Antibacterial and Anti-inflammatory Activities with Low Cytotoxicity and Mechanism of Action in Escherichia coli. *Antimicrob Agents Chemother* **2016,** *60* (1), 495-506.

7. Pandey, B. K.; Srivastava, S.; Singh, M.; Ghosh, J. K., Inducing toxicity by introducing a leucine-zipper-like motif in frog antimicrobial peptide, magainin 2. *Biochem J* **2011,** *436* (3), 609-20.

8. Pires, M. M.; Przybyla, D. E.; Chmielewski, J., A metal-collagen peptide framework for three-dimensional cell culture. *Angew Chem Int Ed Engl* **2009,** *48* (42), 7813-7.

9. Pires, M. M.; Chmielewski, J., Self-assembly of collagen peptides into microflorettes via metal coordination. *J Am Chem Soc* **2009,** *131* (7), 2706-12.

10. Lee, S. G.; Lee, J. Y.; Chmielewski, J., Investigation of pH-dependent collagen triple-helix formation. *Angew Chem Int Ed Engl* **2008,** *47* (44), 8429-32.

11. Thomas, J.; Sharma, A.; Panwar, V.; Chopra, V.; Ghosh, D., Polysaccharide-Based Hybrid Self-Healing Hydrogel Supports the Paracrine Response of Mesenchymal Stem Cells. *ACS Appl Bio Mater* **2019,** *2* (5), 2013-2027.

12. Mukherjee, N.; Ghosh, S.; Sarkar, J.; Roy, R.; Nandi, D., Amyloid-Inspired Engineered Multidomain Amphiphilic Injectable Peptide Hydrogel horizontal line An Excellent Antibacterial, Angiogenic, and Biocompatible Wound Healing Material. *ACS Appl Mater Interfaces* **2023,** *15* (28), 33457-33479.

13. Chang, R.; Yuan, C.; Zhou, P.; Xing, R.; Yan, X., Peptide Self-assembly: From Ordered to Disordered. *Acc Chem Res* **2024,** *57* (3), 289-301.

14. Pal, S.; Sayeed, M.; Kumar, A.; Verma, D. P.; Harioudh, M. K.; Verma, N. K.; Porwal, K.; Sharma, S.; Kulkarni, C.; Bandyopadhyay, A.; Mugale, M. N.; Mitra, K.; Ghosh, J. K.; Chattopadhyay, N., Self-Assembling Nano-Globular Peptide from Human Lactoferrin Acts as a Systemic Enhancer of Bone Regeneration: A Novel Peptide for Orthopedic Application. *ACS Appl Mater Interfaces* **2021,** *13* (15), 17300-17315.

15. Liu, Y.; Zhang, L.; Wei, W., Effect of noncovalent interaction on the self-assembly of a designed peptide and its potential use as a carrier for controlled bFGF release. *Int J Nanomedicine* **2017,** *12*, 659-670.

16. O'Leary, L. E.; Fallas, J. A.; Bakota, E. L.; Kang, M. K.; Hartgerink, J. D., Multi-hierarchical self-assembly of a collagen mimetic peptide from triple helix to nanofibre and hydrogel. *Nat Chem* **2011,** *3* (10), 821-8.

17. Pal, V. K.; Roy, S., Cooperative Metal Ion Coordination to the Short Self-Assembling Peptide Promotes Hydrogelation and Cellular Proliferation. *Macromol Biosci* **2022,** *22* (5), e2100462.

18. Luo, J.; Tong, Y. W., Self-assembly of collagen-mimetic peptide amphiphiles into biofunctional nanofiber. *ACS Nano* **2011,** *5* (10), 7739-47.

19. Jacob, R. S.; George, E.; Singh, P. K.; Salot, S.; Anoop, A.; Jha, N. N.; Sen, S.; Maji, S. K., Cell Adhesion on Amyloid Fibrils Lacking Integrin Recognition Motif. *J Biol Chem* **2016,** *291* (10), 5278-98.

20. Khew, S. T.; Yang, Q. J.; Tong, Y. W., Enzymatically crosslinked collagen-mimetic dendrimers that promote integrin-targeted cell adhesion. *Biomaterials* **2008,** *29* (20), 3034-45.
